# Recognizing senescent vascular smooth muscle cells in diabetic atherosclerosis: biomarkers, transcriptomic signatures, and methodological validity

**DOI:** 10.3389/fendo.2026.1997338

**Published:** 2026-10-30

**Authors:** Xiaoxu Yang, Di Zhao

**Affiliations:** 1 The First Affiliated Hospital, College of Clinical Medicine, Henan University of Science and Technology, Luoyang, China; 2 Luoyang Key Laboratory of Cardiovascular Science, The First Affiliated Hospital, Henan University of Science and Technology, Luoyang, China

**Keywords:** biomarkers, cellular senescence, diabetic atherosclerosis, methodological validity, transcriptomic signatures, vascular smooth muscle cells

## Abstract

Diabetic atherosclerosis represents the most severe macrovascular complication of diabetes mellitus and a major contributor to global morbidity and mortality. Senescence of vascular smooth muscle cells (VSMCs) has been increasingly recognized as a central mechanism driving plaque formation, progression, and instability under diabetic conditions. Yet identifying senescent VSMCs reliably remains difficult: available biomarkers lack specificity, transcriptomic signatures vary considerably across studies, and no consensus has emerged on how senescence should be verified methodologically. This review consolidates current knowledge on senescent VSMC identification in diabetic atherosclerosis. We begin with the pathobiological routes through which hyperglycemia drives VSMC senescence, including oxidative stress, DNA damage, mitochondrial dysfunction, and metabolic reprogramming. We then present a multidimensional classification of senescence-associated biomarkers, spanning cell cycle regulators, senescence-associated β-galactosidase (SA-β-gal), telomere-related markers, the senescence-associated secretory phenotype (SASP), calcification markers, and emerging non-coding RNA signatures, and assess the applicability and shortcomings of each. Transcriptomic signatures derived from single-cell RNA sequencing, spatial transcriptomics, and flow cytometry are then integrated to map the heterogeneity of senescent VSMC subpopulations and their functional roles within atherosclerotic plaques. We next appraise the methodological validity of existing detection strategies, comparing *in vitro* high-glucose models, *in vivo* diabetic animal models, and clinical specimens, and trace the sources of inter-study inconsistency, including spatial heterogeneity, cross-sectional designs, and the absence of standardized protocols. Finally, we outline a stepwise identification pipeline extending from molecular markers to functional validation and multi-omics integration, and consider future prospects such as senolytic therapies and targeted delivery systems. By providing this methodological reference, we hope to support both basic research and clinical translation and to push the field toward more precise, standardized identification of senescent VSMCs.

## 1 Introduction

Diabetic atherosclerosis runs a considerably more complex pathological course than non-diabetic disease. Hyperglycemia, the defining metabolic feature of diabetes, accelerates vascular cell aging; the senescence of VSMCs that follows is increasingly regarded as a core driver of plaque instability and calcification. Elevated glucose promotes VSMC senescence and osteoblast-like transdifferentiation through multiple routes, ultimately fueling vascular calcification (VC) and atherosclerotic progression. High-glucose conditions shift VSMCs toward a pronounced senescence-associated phenotype marked by proliferative arrest, a secretory phenotypic switch, and apoptosis resistance. High glucose, for example, upregulates p21 and expands the fraction of senescence-associated β-galactosidase (SA-β-gal)-positive cells (1). Combined high-glucose and high-phosphate stimulation worsens VSMC senescence and calcification by repressing SIRT1 and activating NF-κB signaling (2). High glucose also triggers VSMC calcification through endoplasmic reticulum (ER) stress and apoptosis, an effect that the ER stress inhibitor 4-PBA substantially blunts (3). Collectively, these data show that hyperglycemia drives VSMC senescence through multiple pathways, such as the SIRT1/NF-κB axis and ER stress, which together underpin plaque instability and calcification in diabetic atherosclerosis.

A natural question is whether VSMC senescence is unique to diabetic atherosclerosis or also occurs in non-diabetic disease. It is now well established that senescent VSMCs are present in the arterial wall during normal aging and in non-diabetic atherosclerotic plaques. Matthews et al. (4) demonstrated telomere-based senescence of VSMCs in human atherosclerotic plaques from non-diabetic patients, and Wang et al. (5) showed in ApoE−/− mice that VSMC senescence promotes atherogenesis even in the absence of hyperglycemia. Grootaert et al. (6) further reviewed that VSMC death, autophagy, and senescence are general features of plaque progression. These findings indicate that the core senescence machinery—p53-p21 and p16-Rb activation, SASP secretion, and growth arrest—is not diabetic-specific.

Whether diabetic and non-diabetic VSMC senescence can be reliably distinguished *in vivo*, however, remains less clear. No study has yet performed a direct head-to-head comparison of senescent VSMCs between diabetic and non-diabetic plaques using identical markers and single-cell resolution, and most published work examines either diabetic or non-diabetic models separately. Indirect approximations are nonetheless possible: parallel reading of studies using streptozotocin (STZ)-induced or db/db diabetic models versus ApoE−/− or LDLR−/− non-diabetic atherosclerotic models allows rough cross-study comparison of p16, p21, and senescence-associated β-galactosidase (SA-β-gal) positivity rates. Immunohistochemical comparisons of senescence marker intensity and plaque localization between diabetic and non-diabetic human plaque cohorts provide another window, and cross-study juxtaposition of single-cell transcriptomic datasets from diabetic versus non-diabetic plaques offers yet another, although differences in clustering algorithms and senescence gene-set selection limit their direct comparability. Taken together, although direct head-to-head comparisons remain lacking, the available evidence suggests that senescence markers tend to be more abundant and to appear earlier in diabetic plaques.

A further question is whether diabetic VSMC senescence represents a quantitatively enhanced process or a qualitatively distinct biological state. Arguments for quantitative enhancement rest on the observation that the core effector machinery is shared—p53-p21, p16-Rb, and NF-κB-driven SASP operate in both diabetic and non-diabetic VSMC senescence (6, 7)—and that cross-study comparisons suggest that senescence markers tend to be more abundant and to appear earlier in diabetic plaques. At the same time, several features appear diabetic-specific rather than merely amplified. Metabolic memory, in which epigenetic marks induced by early hyperglycemia persist even after glycemic control, is a diabetes-specific feature of vascular aging (8). Accelerated telomere attrition driven by chronic oxidative stress and advanced glycation end-product (AGE) accumulation exceeds what would be expected from chronological aging alone (5, 9). Altered SASP composition under high-glucose conditions, with a pro-inflammatory and pro-calcific bias more pronounced than in non-diabetic senescence, further points to qualitative distinctness (7, 10). The current reading of the literature is therefore that the basal senescence program is shared (quantitative enhancement), but that diabetes superimposes additional, qualitatively distinct layers—particularly epigenetic metabolic memory—on top of it. This remains an open question that future side-by-side comparison studies, with matched senescence panels and longitudinal sampling before, during, and after glycemic control, will need to resolve definitively.

A related translational question is whether senescent VSMCs have been conclusively demonstrated in human diabetic atherosclerotic plaques. The evidence is currently indirect rather than definitive. On the one hand, Matthews et al. (4) demonstrated telomere-based senescence of VSMCs in human atherosclerotic plaques—including upregulation of p16 and SA-β-gal activity—but this study did not stratify patients by diabetes status. On the other hand, Hemmings et al. (11) showed that VSMCs cultured from type 2 diabetic patients exhibit persistently elevated DNA damage markers and senescence features, together with microRNA-145 upregulation and SASP-related cytokine secretion, compared with VSMCs from non-diabetic donors. Single-cell RNA sequencing studies of human plaques have identified “senescent-like” VSMC subpopulations (12), but most published datasets combine diabetic and non-diabetic plaques or do not report diabetes-stratified analyses. It is therefore fair to say that senescent VSMCs have been demonstrated in human atherosclerotic plaques, and that diabetic VSMCs show enhanced senescence characteristics ex vivo, but a conclusive, diabetes-specific demonstration of senescent VSMCs within human diabetic plaques—using matched senescence panels and diabetes-stratified sampling—remains to be performed. This gap underscores the translational relevance of the marker framework proposed in this review: standardized, diabetes-stratified plaque studies are needed to confirm the prevalence and functional role of senescent VSMCs in human diabetic atherosclerosis.

Senescent VSMCs are characterized by proliferative arrest, a secretory phenotypic switch, and resistance to apoptosis, but recognizing them *in situ* is far from straightforward. No single marker is unique to senescence, so identifying these cells requires a combination of indicators. Current practice relies chiefly on SA-β-gal staining, cyclin-dependent kinase inhibitors such as p21 and p16, and osteogenic markers such as Runx2 and osteopontin (OPN). Each of these signals, however, can appear in non-senescent settings, and their expression differs with experimental model and pathological state. In high-glucose-induced VSMC calcification/senescence, for instance, the long non-coding RNAs lncRNA-ES3 and SNHG1 shift markedly in expression: lncRNA-ES3 operates through Bhlhe40 and the miR-95-5p/miR-6776-5p axis, whereas SNHG1 acts via Bhlhe40 and Atg10-dependent autophagy (13, 14). The BMF-AS1/BMF axis also participates in this process, and plasma BMF levels in diabetic patients track positively with coronary artery calcification scores (15). Taken together, these observations argue that a single molecular readout cannot capture the full complexity of the senescent VSMC state; evaluation should instead integrate indicators at several levels, including SASP output, autophagic activity, and mitochondrial function. A biomarker-based identification system assembled from multiple complementary readouts is therefore essential for accurate detection of senescent VSMCs in diabetic atherosclerosis.

High-throughput platforms such as single-cell RNA sequencing (scRNA-seq) have made it possible to characterize the genome-wide transcriptional landscape of senescent VSMCs, opening new avenues for uncovering their heterogeneity and for discovering new markers (12, 16). At single-cell resolution, scRNA-seq distinguishes cell subpopulations and rare states that bulk approaches often miss (17, 18). Within the diabetic atherosclerotic milieu, high glucose reprograms VSMC metabolism, dysregulating the hexosamine biosynthetic pathway and the pentose phosphate pathway (19, 20), and pushes cells toward osteo-/chondrogenic phenotypes (21); NLRP3/IL-1β/NF-κB-linked inflammatory cascades further accelerate atherosclerosis (22, 23). Spatial proteomics and RNA sequencing have likewise exposed a role for Nup93-dependent aberrant RNA splicing: when VSMCs are exposed to combined high glucose and ox-LDL, Nup93 declines, nuclear import of SRSF1 and SRSF3 is impaired, and SerpinE2 splicing goes awry, fueling VSMC proliferation (24). A mitochondria-targeted natural antioxidant nanosystem (T4O@TPP/PEG-PLGA) has also been proposed as a candidate for mitigating diabetic vascular calcification: the nanodrug reduces high-glucose-induced oxidative stress in VSMCs and, in diabetic vascular calcification models, mitigates calcification and restores mitochondrial morphology (25).

Despite this progress, divergent experimental designs, senescence induction protocols, and validation strategies limit how far results can be compared across studies, and methodological validity has emerged as a concern that demands systematic evaluation and standardization. Experimental approaches to model VSMC senescence in diabetic atherosclerosis include *in vitro* high-glucose exposure (e.g., 30 mM glucose) (9) and streptozotocin (STZ)-induced diabetic mice *in vivo* (26). Glucose concentration, treatment duration, cell source (human aortic VSMCs vs. mouse VSMCs), and the induction regimen (high glucose alone or combined with high phosphate or ox-LDL) all vary widely, and these differences can yield conflicting findings. Some reports, for example, find that high glucose does not directly modulate high-phosphate-driven VSMC phenotypic switching or medial arterial calcification (27), whereas others describe a clear synergistic interaction (2). Senescence assessment is similarly unstandardized: some studies rely on SA-β-gal staining, the classical senescence marker (28), while others quantify p21, p16, or γ-H2AX as readouts of cell-cycle arrest and DNA damage (9, 12, 29). *In vivo*, STZ-induced diabetes and genetic models such as db/db mice represent different diabetes models, adding further cross-study variability (26, 30). Standardized protocols (unified induction conditions, multi-marker readouts, and cross-validation of *in vitro* and *in vivo* findings) are thus needed to make results more comparable and reproducible.

Recent reviews have surveyed cellular senescence in cardiovascular disease and its therapeutic targeting (31). The present review is deliberately narrower and method-oriented: it brings together studies on senescent VSMC identification specifically in diabetic atherosclerosis along three axes (biomarkers, transcriptomics, and methodology) and offers the field an evidence map and practical recommendations. On the biomarker axis, we concentrate on hyperglycemia-associated molecular markers of VSMC senescence, including lncRNA-ES3 and miR-34c-5p, whose involvement in high-glucose-induced VSMC calcification/senescence has been demonstrated in primary studies (14, 32); calcification markers such as osteopontin also rise in VSMCs under high-glucose conditions (21), and high glucose potentiates IL-1β-driven inflammatory responses in VSMCs (22). On the transcriptomic axis, we draw on scRNA-seq and spatial transcriptomic data to construct a signature map of senescent VSMCs and to probe their heterogeneity and functional links. On the methodological axis, we weigh the strengths and weaknesses of different senescence induction models (e.g., high glucose, high phosphate, and oxidative stress), dissect the biases present in the existing literature, and put forward standardization recommendations. Through this synthesis, we aim to provide a comprehensive evidence base for identifying senescent VSMCs in diabetic atherosclerosis and to orient future basic research and clinical translation.

**Figure 1** outlines the biological drivers, phenotypic consequences, and identification framework of senescent VSMCs in diabetic atherosclerosis.

**Figure 1:**
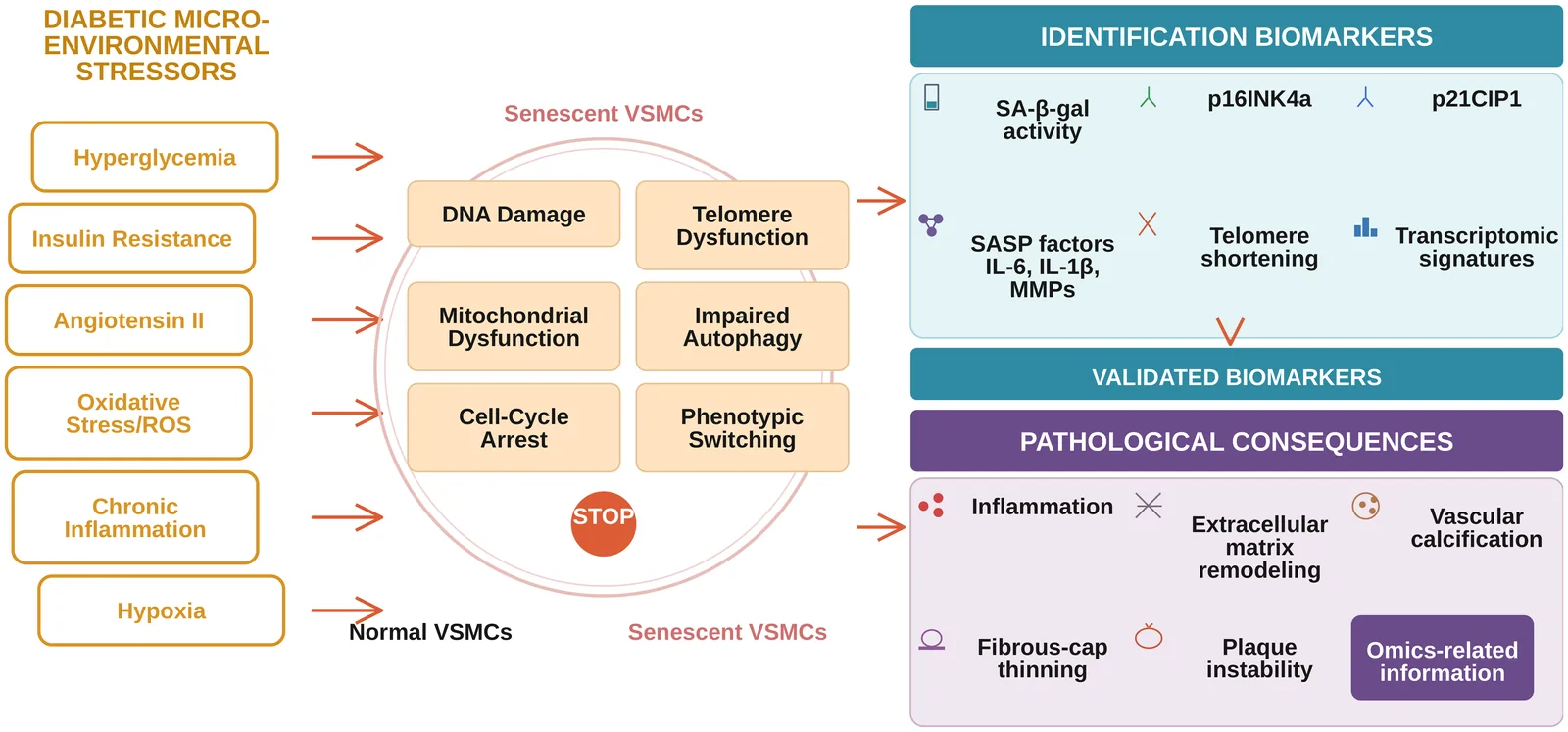
Biological drivers, phenotypic consequences, and identification framework of senescent vascular smooth muscle cells (VSMCs) in diabetic atherosclerosis. Diabetic microenvironmental stressors (hyperglycemia, insulin resistance, angiotensin II, oxidative stress/ROS, chronic inflammation, and hypoxia) drive normal VSMCs into senescence through DNA damage, telomere dysfunction, mitochondrial dysfunction, impaired autophagy, cell-cycle arrest, and phenotypic switching. Identification biomarkers (SA-β-gal activity, p16INK4a, p21CIP1, SASP factors, telomere shortening, and transcriptomic signatures) and pathological consequences (inflammation, extracellular matrix remodeling, vascular calcification, fibrous-cap thinning, and plaque instability) are indicated. ROS, reactive oxygen species; SA-β-gal, senescence-associated β-galactosidase; SASP, senescence-associated secretory phenotype; VSMC, vascular smooth muscle cell.

## 2 Pathobiological basis of VSMC senescence in diabetic atherosclerosis

### 2.1 Molecular mechanisms of high-glucose-induced VSMC senescence

High-glucose conditions push VSMCs into senescence (9) through interwoven signaling pathways: high glucose increases ROS production in VSMCs (33) and induces DNA damage with enhanced senescence (29). Under diabetic conditions, sustained hyperglycemia increases ROS production in VSMCs, driven by polyol pathway overactivity (34), AGE receptor-mediated oxidant stress (35), and PKC-dependent NAD(P)H oxidase activation (33). AGEs (non-degradable protein modifications formed under hyperglycemia) cross-link extracellular matrix components and, through binding to cell-surface receptors, ignite multiple pro-inflammatory cascades that worsen redox imbalance (36). The antioxidant capacity itself deteriorates with age: carotid arteries of aged rhesus macaques carry elevated 8-iso-PGF2α and 4-HNE, while induction of the antioxidant transcription factor Nrf2 and its targets (NQO1, GCLC, HMOX1) fails; this Nrf2 insufficiency amplifies oxidative damage in VSMCs challenged with high glucose and favors NF-κB-driven vascular inflammation (37). High glucose additionally induces VSMC senescence by raising thioredoxin-interacting protein (TXNIP), whose transcriptional activator carbohydrate-responsive element-binding protein (ChREBP) promotes p53 activation by suppressing AKT phosphorylation, producing a senescence phenotype that feeds atherosclerotic progression (38). DNA damage signaling matters equally here: in rats subjected to diabetes plus balloon injury, senescent vascular cells stain positively for γ-H2AX, phospho-ATM, phospho-CHK2, and phospho-p53, pointing to DNA double-strand break repair in diabetes-accelerated atherosclerosis (39). Together, high-glucose-evoked oxidative stress and DNA damage activate the senescence-associated secretory phenotype (SASP), with cell cycle inhibitors such as p21 and p16 upregulated and pro-inflammatory cytokines and matrix-degrading enzymes released in excess, creating an environment in which plaque formation and progression thrive (40).

Mitochondrial dysfunction sits at the core of high-glucose-induced VSMC senescence. In diabetes, mitochondria serve simultaneously as the main ROS source and the chief target of oxidative injury, creating a self-reinforcing cycle. Primary cerebrovascular smooth muscle cells (CVSMCs) treated with high glucose show elevated mitochondrial superoxide, reduced ATP output, impaired respiration, depolarized membranes, and imbalanced dynamics: fusion protein OPA1 falls while fission protein MFF rises, fragmenting the mitochondrial network (41). The Slc25a12 mitochondrial carrier, which exchanges aspartate/glutamate across the inner membrane, declines sharply after high-glucose injury; its loss drives mitochondrial dysfunction and senescence, whereas the TRPV1 agonist capsaicin restores function by raising Slc25a12 (42). Exosomes released by high-glucose-stimulated endothelial cells also deliver versican (VCAN) to VSMCs, lowering mitochondrial membrane potential and the expression of HADHA and Cox-4, and in doing so induce calcification and senescence (43). Excess mitochondrial ROS further attacks mitochondrial DNA, promoting mutation accumulation and respiratory chain failure while hastening telomere shortening; reduced telomerase activity is a hallmark of high-glucose-induced VSMC senescence, and adiponectin counteracts it by activating AMPK/TSC2/mTOR/S6K1 while restraining PI3K/Akt/mTOR/S6K1 (9). Mitochondrial dysfunction and NLRP3 inflammasome activation are tightly coupled: FGF21 overexpression relieves VSMC senescence in diabetic mice by dampening SYK phosphorylation, NLRP3 dimer formation, and its colocalization with ASC and caspase-1, restoring PPARγ and catalase (CAT) levels (44). Sodium tanshinone IIA sulfonate exerts similar anti-aging effects through the A20-NF-κB-NLRP3-CAT axis (45). On balance, mitochondrial dysfunction is not merely an early event in high-glucose-induced VSMC senescence; it is a central hub that drives the senescence program irreversibly forward.

Diabetes-associated metabolic memory offers a useful framework for understanding why VSMC senescence persists: in type 2 diabetic db/db mice, diabetes-induced SMC changes were not reversed by glycemic control (30). Even after effective glycemic control is achieved, loss of the contractile VSMC phenotype and associated chromatin changes persist, implying that molecular changes triggered by prior hyperglycemia exert long-lasting effects (30). In VSMCs from diabetic mice, several microRNA expression changes survive *in vitro* culture, suggesting that epigenetic modifications form the molecular basis of this memory (46). miR-504, for example, is markedly upregulated in VSMCs of db/db diabetic mice; its targets Grb10 and Egr2 regulate growth factor signaling, and miR-504 overexpression suppresses contractile gene expression while boosting ERK1/2 activation, proliferation, and migration, changes that endure in culture (46). High-glucose-induced VSMC senescence (9) likewise entails persistent molecular alterations (30), including elevated O-GlcNAcylation of VSMC proteins (47). O-GlcNAcylation, a post-translational modification that adds N-acetylglucosamine to serine or threonine residues (47), modifies target proteins through the hexosamine biosynthetic pathway using the glucose metabolite UDP-GlcNAc; hyperglycemia elevates O-GlcNAc transferase activity and alters the pattern of O-GlcNAcylated proteins in VSMCs (47), and OGT-mediated O-GlcNAcylation of MAGI1 promotes VSMC dysfunction—including proliferation, dedifferentiation, and inflammatory responses—under high-glucose conditions via the PI3K/AKT pathway (48). O-GlcNAcylation also drives VSMC osteogenic differentiation: OGT-mediated O-GlcNAcylation of RUNX2 advances the osteogenic differentiation of human coronary artery smooth muscle cells and aggravates diabetic vascular calcification (49); consistently, O-GlcNAcylation promotes VSMC osteogenic differentiation and calcification in diabetes (50). Metabolic memory also manifests as mitochondrial DNA damage: in diabetic vascular calcification models, mitochondrial DNA damage is aggravated in a reactive oxygen species-dependent manner and contributes to VSMC senescence (51). Senescent cells then propagate senescence signals to neighbors in a paracrine fashion: aberrant STING activation in macrophages suppresses autophagy through ULK1 (S757) phosphorylation, accelerating macrophage senescence and, via SASP, enhancing VSMC senescence in co-culture, thereby creating a tissue-level senescence network (52). This helps explain why vascular senescence continues after glycemia normalizes, and it implies that intervening before metabolic memory is entrenched may outperform acting after the senescence program has begun. The BMF-AS1/BMF axis, upregulated in diabetic mice and patients in proportion to vascular calcification, illustrates how early targeting of such pathways might carry therapeutic promise (15).

### 2.2 Diabetic-specific stressors driving VSMC senescence

#### 2.2.1 Advanced glycation end products and RAGE signaling

Advanced glycation end products (AGEs) and their receptor RAGE constitute a diabetic-specific axis that drives VSMC senescence beyond the general effects of hyperglycemia. AGEs form non-enzymatically when persistent hyperglycemia reacts with proteins and lipids, and their accumulation in the vascular wall activates RAGE, a pattern-recognition receptor whose engagement triggers NF-κB-dependent inflammatory signaling and ROS overproduction (36, 53). In VSMCs, AGE exposure has been shown to accelerate p53-p21 pathway activation and increase SA-β-gal positivity, while also promoting osteogenic differentiation through Runx2 upregulation (53, 54). A particularly important feature of this pathway is its self-amplifying nature: AGE-induced oxidative stress further accelerates AGE formation, creating a positive feedback loop that sustains vascular injury even after glycemic control improves (36). This slow turnover and self-propagating capacity make AGE-RAGE signaling one mechanistic link between metabolic memory and sustained VSMC senescence in diabetic atherosclerosis.

#### 2.2.2 Insulin resistance and VSMC senescence

Insulin resistance adds a parallel, hyperglycemia-independent route to VSMC senescence. Under normal conditions, insulin signaling through the PI3K-AKT pathway supports VSMC survival and suppresses pro-apoptotic signaling; in insulin-resistant states this protective arm is impaired, shifting VSMCs toward a synthetic, pro-inflammatory phenotype (55). Martínez-Hervás et al. (55) demonstrated that insulin resistance reduces VSMC survival and increases CX3CL1/CX3CR1 axis activity, aggravating atherosclerotic lesion formation. The coexistence of hyperglycemia and insulin resistance in type 2 diabetes therefore creates two concurrent senescence-driving inputs—metabolic and mitogenic—that are not fully captured by hyperglycemia-only *in vitro* or *in vivo* models.

#### 2.2.3 Mitochondrial dysfunction

Mitochondrial dysfunction sits at the core of high-glucose-induced VSMC senescence. In diabetes, mitochondria serve simultaneously as a major source of ROS and a target of oxidative injury, creating a self-reinforcing cycle (56, 57). Persistent hyperglycemia has been associated with increased mitochondrial electron leak and superoxide production, which can damage mitochondrial DNA and impair respiratory chain function (51). In VSMC calcification models, p53 has been shown to directly regulate mitochondrial dynamics, linking cell-cycle arrest to structural mitochondrial remodeling (57). Pharmacological interventions that improve mitochondrial function—such as mitochondria-targeted antioxidants—can reduce high-glucose-induced VSMC senescence and attenuate vascular calcification, confirming the causal role of mitochondrial dysfunction in this process (25, 51).

#### 2.2.4 Epigenetic mechanisms and metabolic memory

Diabetes-associated metabolic memory offers a useful framework for understanding why VSMC senescence persists even after glycemic control is achieved. Epigenetic changes initiated by early hyperglycemic exposure—including DNA methylation, histone modifications, and O-GlcNAcylation—can persist long-term, sustaining the expression of pro-inflammatory and pro-senescence genes (8, 58). El-Osta et al. (8) demonstrated that transient high glucose causes persistent epigenetic changes and altered gene expression during subsequent normoglycemia, providing direct experimental evidence for metabolic memory in vascular cells. In VSMCs, elevated O-GlcNAc modification seen in diabetic arteries tracks with aggravated vascular calcification and osteogenic differentiation (59, 60). Non-coding RNAs add another layer of epigenetic regulation: lncRNA-ES3 is upregulated in high-glucose-induced VSMC calcification and, acting as a competitive endogenous RNA, sponges miR-34c-5p to relieve inhibition of the target gene BMF and favor the senescence phenotype (14, 32). miR-34a, by contrast, is upregulated in aged vessels and has been implicated in VSMC senescence and osteogenic calcification, in part through downregulation of SIRT1 (61, 62). These epigenetic mechanisms collectively help explain why VSMC senescence in diabetes is not simply reversed when glucose levels are normalized.

### 2.3 Contribution of senescent VSMCs to atherosclerotic plaque progression

Senescent VSMCs reshape the plaque microenvironment by secreting pro-inflammatory cytokines, chemokines, and matrix-remodeling enzymes, collectively the senescence-associated secretory phenotype (SASP), and this activity drives atherosclerotic progression. They release abundant IL-1α, which acts in an autocrine loop to induce downstream cytokines and chemokines, attracting monocytes and intensifying intra-plaque inflammation (31). Matrix metalloproteinase-9 (MMP-9) activity rises while collagen secretion falls, directly undermining the fibrous cap’s mechanical strength and predisposing it to rupture (31). Beyond recruiting inflammatory cells, the SASP of senescent VSMCs sustains vascular inflammation and accelerates atherosclerotic lesion progression (63), fueling a self-reinforcing inflammatory cycle within the plaque. Extracellular vesicles shed by senescent VSMCs (senEVs) form an integral part of SASP: their cargo proteome (e.g., SERPINF1 and THBS1) modulates T-cell and monocyte functions and elicits pro-inflammatory cytokine secretion, further amplifying immune-inflammatory responses within the plaque (32). Senescent VSMCs, then, are hardly passive occupants of the plaque; through SASP they actively build up and propagate the inflammatory microenvironment, promoting plaque instability.

While the SASP and extracellular vesicle-mediated signaling from senescent VSMCs have been described (see Section 3.1), the bidirectional nature of this communication network deserves emphasis. Most existing studies focus on one direction—endothelium-to-VSMC or macrophage-to-VSMC signaling—but recent evidence supports a model in which senescent VSMCs actively contribute to plaque inflammation through paracrine effects on neighboring cell types. On the macrophage side, the SASP secreted by senescent VSMCs includes chemokines and pro-inflammatory cytokines such as IL-6 and CXCL8 (64), which can contribute to monocyte recruitment and maintenance of the inflammatory plaque milieu. On the endothelial side, hyperglycemia-stimulated endothelial cells deliver pro-senescence cargo to VSMCs through extracellular vesicles—including exosomal Notch3 (1), versican-containing exosomes (43), and circRNA-0077930 carried by endothelial exosomes (65)—and senescent VSMCs themselves secrete exosomes with pro-atherosclerotic cargo under diabetic conditions (66). Unbiased proteomic analysis of extracellular vesicles secreted by senescent human VSMCs further reveals their capacity to modulate neighboring cell behavior (67). This emerging evidence suggests that VSMC senescence in diabetes is embedded within an intercellular communication network rather than being a purely cell-autonomous state, and that diabetes amplifies this signaling through both extracellular vesicle-mediated transfer and soluble SASP factors.

Sex-related differences in VSMC senescence remain an understudied area, yet existing evidence suggests that biological sex may modulate both the magnitude and the molecular pathways of senescence in diabetic atherosclerosis. Estrogen has been shown to delay arterial senescence in mice (68), and sex-specific effects of dietary interventions on vascular senescence and plaque burden have been reported in ApoE−/− mice (69), with sexual dimorphism also documented in atherosclerotic plaques of aged Ldlr−/− mice (70); together with broader evidence that biological sex shapes vascular aging and diabetic cardiovascular disease (61, 71), these findings indicate that female and male VSMCs may respond differently to pro-senescence stressors. Despite this, most published studies of VSMC senescence in diabetic atherosclerosis do not report sex-stratified analyses, and it remains unclear whether the senescence markers, SASP composition, or therapeutic response to senolytics differ between female and male patients. This gap is clinically relevant because women with diabetes experience disproportionately elevated cardiovascular risk compared with men (72), and sex-specific mechanisms of VSMC senescence may contribute to this disparity. Future studies with balanced sex cohorts and sex-stratified reporting are needed to determine whether VSMC senescence in diabetic atherosclerosis is a sexually dimorphic process.

In diabetic atherosclerosis, VSMC senescence and enhanced osteoblast-like differentiation are key cellular drivers of accelerated plaque calcification (49, 51). Senescent VSMCs raise the osteogenic transcription factor Runx2 and osteocalcin while repressing smooth muscle markers such as SM22α, a clear osteoblast-like switch (73, 74). This switch is intimately tied to senescence itself. IL-1β, for example, provokes premature VSMC senescence via the NF-κB/p53/p21 pathway and nudges cells toward an osteoblast-like fate, culminating in vascular calcification (74). Senescent macrophages add to the burden by secreting interferon-induced transmembrane protein 3 (IFITM3), which accelerates VSMC calcification and senescence (73). AGEs, which are increased in diabetes (53), likewise foster VSMC senescence and calcification: in an *in vitro* VSMC calcification model, the AGE carboxymethyl-lysine (CML) aggravates mitochondrial DNA damage in a reactive oxygen species-dependent manner and contributes to VSMC senescence-associated calcification (51). Senescent VSMCs thus link cellular senescence to plaque calcification (51); in the diabetic context, O-GlcNAcylation promotes VSMC osteogenic differentiation and calcification (50), and diabetes-induced vascular calcification is accompanied by enhanced stiffness (50).

The build-up of the cell cycle inhibitors p16Ink4a and p21Cip1 in VSMCs plays a key role in plaque progression and necrotic core expansion (4, 56, 75, 76). In human atherosclerotic plaques, VSMCs in the fibrous caps accumulate p16 and p21 together with senescence-associated β-galactosidase (SA-β-gal) activity, and telomere shortening increases with atherosclerotic severity (4), directly linking the accumulation of these cell cycle inhibitors in plaque VSMCs to progressive disease in patients. Sustained expression of these inhibitors locks VSMCs into irreversible growth arrest, depriving the plaque of cells capable of repairing vascular injury and maintaining fibrous cap integrity, and thereby contributing to plaque progression and instability (4). Causally, p21-dependent premature senescence of VSMCs promotes atherogenesis: angiotensin II induces VSMC senescence via the p53/p21 pathway and elevates p21 in atherosclerotic vessels, whereas loss of p21 abrogates angiotensin II-induced vascular inflammation and lesion development (77). Consistently, VSMC senescence itself promotes atherosclerosis and features of plaque vulnerability: plaque VSMCs show reduced expression of the telomere-protective protein TRF2, and VSMC-specific expression of a dysfunctional TRF2 mutant increases atherosclerosis and necrotic core formation in mice, whereas VSMC-specific TRF2 increases the relative fibrous cap and decreases necrotic core area (5). p16- and p21-mediated arrest is therefore both a hallmark of VSMC senescence and a cellular basis for plaque transition from stability toward instability and necrotic core expansion.

## 3 Classical biomarker systems for senescent VSMCs

### 3.1 Cell cycle regulation-related markers

Disrupted cell cycle regulation is a defining molecular feature of senescent VSMCs, and the associated marker system extends from cyclin-dependent kinase inhibitors to proliferation proteins. p16Ink4a, a cyclin-dependent kinase inhibitor, remains a widely used senescence biomarker. Its expression accumulates in the arterial wall during vascular aging and tracks closely with telomere shortening and DNA damage responses (4); epigenetic remodeling likewise contributes to vascular senescence (58). The arrest mediated by p16Ink4a is enforced through specific inhibition of cyclin D–CDK4 complexes (78); in an Rb-dependent manner, it keeps the retinoblastoma protein (Rb) hypophosphorylated so that cells are locked in G1 (79). Yet p16Ink4a is also detected in non-senescent cells: it is expressed in proliferating normal T lymphocytes (80), and can be induced reversibly in macrophages as part of a physiological immune response (81). Moreover, genetic background shapes its expression (75, 76). Relying on p16Ink4a alone to identify senescent VSMCs therefore demands caution; combining several markers is preferable.

p21Cip1, a principal downstream effector of the p53 pathway (82), mainly mediates DNA damage-induced senescence and is of particular relevance in the diabetic vascular niche. It blocks the G1/S checkpoint by suppressing cyclin E-CDK2 and cyclin A-CDK2 kinase activity (83), and its expression is controlled by both p53-dependent and p53-independent routes (82, 84). In the diabetic vasculature, hyperglycemia engages this axis directly: high glucose induces premature VSMC senescence with upregulation of both p21 and p53 (9), and TXNIP, upregulated by high glucose, suppresses AKT phosphorylation and leads to p53 activation and VSMC senescence (38). Beyond these core regulators, circular RNAs add a further layer of control by sponging miRNAs: circRNA-0077930 delivered by endothelial exosomes under hyperglycemic conditions acts as a miR-622 sponge, up-regulating p21, p53 and p16 in VSMCs and driving their senescence (65), thereby enriching the regulatory network of diabetic vascular senescence.

Positive cell cycle regulators offer corroborative evidence beyond these core inhibitors. Rb phosphorylation status is a key switch in cell cycle progression: the retinoblastoma protein constrains the G1-S transition, and phosphorylation of pRb in G1 inactivates its growth-inhibitory function, allowing cell cycle progression after sequential modification by distinct cyclin-CDK complexes (85); CDK phosphorylation at specific sites inactivates the individual binding domains of Rb (86). Conversely, in senescent cells the growth arrest is enforced by cyclin-dependent kinase inhibitors such as p21WAF1 and p16INK4a (87), and Rb is recruited to E2F-responsive promoters, stably repressing E2F target genes (88). Correspondingly, Ki-67, a proliferation-associated nuclear antigen whose expression is coupled to cell-cycle progression, is largely absent from growth-arrested cells, and its loss—together with the silencing of E2F target genes—provides molecular proof of functional proliferative arrest (88, 89). Senescent and quiescent cells both proliferate slowly, but the underlying states differ fundamentally: quiescent cells resume cycling upon appropriate stimulation, whereas senescent arrest is largely irreversible (87). Pairing Rb phosphorylation status with Ki-67 absence, supplemented by SA-β-gal staining and SASP detection, can therefore separate senescent VSMCs from G0-phase quiescent cells. In diabetic atherosclerosis, where high glucose directly induces premature VSMC senescence (9) and drives VSMC senescence through TXNIP-mediated p53 activation (38), multidimensional assessment built on these cell cycle markers should sharpen the molecular portrait of senescent VSMCs and inform senescence-targeted therapies.

### 3.2 Applicability and limitations of senescence-associated β-galactosidase detection

SA-β-gal staining is the most widely applied gold-standard method for detecting cellular senescence in histology and cultured cells. The assay exploits the markedly enhanced activity of lysosomal β-galactosidase at the suboptimal pH of 6.0 in senescent cells (90). In non-senescent cells the enzyme is most active at pH 4.0; in senescent cells, increased lysosomal content and quality permit efficient hydrolysis of the X-Gal substrate at pH 6.0, yielding an insoluble blue precipitate (91, 92). Applied to frozen sections of vascular tissue, however, the method suffers from a low signal-to-noise ratio: the complex structure of vascular tissue and partial enzyme inactivation during cryosectioning raise background and non-specific staining, complicating interpretation of positive signals (93). In diabetic atherosclerotic plaques, SA-β-gal-positive cells include not only senescent VSMCs but also abundant macrophages and foam cells (39). Establishing that a senescence signal originates from VSMCs therefore requires co-staining with cell-specific markers such as smooth muscle α-actin (SMα-actin) or SM22α. In diabetic mouse aortas, for example, SA-β-gal combined with p21 immunofluorescence colocalization cleanly separates VSMC senescence from that of other cell types (44, 45). Co-labeling of SA-β-gal with S100β has likewise confirmed Schwann-cell-specific SA-β-gal expression after denervation (94), underscoring the need for co-staining to determine cellular origin.

To move past the subjectivity and semi-quantitative nature of conventional SA-β-gal staining, quantitative image analysis systems (e.g., automated threshold segmentation in ImageJ) improve objectivity and reproducibility (92). Unified thresholds that automatically identify and measure positive staining area as a proportion of total area reduce interpretive bias. In diabetic mouse aorta studies, for instance, ImageJ-based quantification showed that both FGF21 overexpression and sodium tanshinone IIA sulfonate treatment significantly shrank SA-β-gal-positive regions (44, 45). Still, no standardized protocol exists in the diabetic atherosclerosis field: staining time, pH, incubation temperature, and image acquisition parameters all differ between studies, hampering direct comparison (93). Some protocols yield satisfactory results with 2 h staining at pH 4.0, while others require overnight incubation at pH 5.8 (93). Cryopreservation duration also matters, with long-term storage (e.g., 4–12 months) attenuating staining signals (93). A standardized SA-β-gal detection and quantification protocol tailored to vascular tissue, especially diabetic atherosclerotic plaques, is therefore needed to ensure comparability and reliability.

### 3.3 Telomere length and telomerase activity assessment

Telomere shortening, a hallmark of replicative senescence, occupies a central place in VSMC senescence during diabetic atherosclerosis. Under high-glucose conditions, oxidative stress and inflammation accelerate telomere DNA attrition and drive VSMCs prematurely into senescence (26). Reduced telomerase activity is a major driver of this shortening: high glucose lowers VSMC telomerase activity by inhibiting the AMPK/TSC2/mTOR/S6K1 pathway while activating PI3K/Akt/mTOR/S6K1 (9). Among detection approaches, qPCR-based telomere length assays (e.g., the Cawthon method) need only minimal DNA and are operationally simple, which suits the minute amounts of DNA extractable from plaque tissue. The catch is that such assays report the average telomere length across all cells in a sample and cannot resolve differences among subpopulations, an important limitation in plaques whose cellular composition is highly heterogeneous.

Aberrant expression of telomere-protective proteins such as TRF2 can supply complementary markers for identifying senescent VSMCs (5). TRF1 and TRF2, core members of the telomere-protective complex, bind telomeric DNA and control telomere length (95); in human plaques, VSMCs show reduced expression and telomere binding of TRF2 (5). In diabetes-associated vascular disease, where VSMC senescence is accelerated by hyperglycemia (9, 38), TRF2 downregulation is associated with increased DNA damage, and TRF2 functional inactivation promotes VSMC senescence and atherosclerosis in experimental models (5). This carries potential clinical significance: declining TRF2 in plaques could serve as an early warning indicator of VSMC senescence and open a therapeutic window in diabetic atherosclerosis (5). Telomerase activity assays (e.g., TRAP-PCR silver staining) further inform senescence assessment, and endogenous protective factors such as adiponectin postpone high-glucose-induced premature VSMC senescence by restoring telomerase activity (9).

Although telomere length measurement has been applied in senescence research, its translation to clinical use encounters several hurdles (4). First, baseline telomere length varies widely among individuals—even among those of the same age—owing to heritable and environmental factors, such variability may limit the sensitivity of cross-sectional comparisons (96). Second, atherosclerotic plaques harbor highly heterogeneous cell populations; single-cell studies reveal VSMC subpopulations that differ in differentiation and senescence states, and tissue-level averages may obscure such key subpopulation differences (12, 17, 18). High-throughput quantitative fluorescence *in situ* hybridization (HT-Q-FISH) has been validated for accurate telomere length measurement in large cell populations; applied to plaques, it could expose telomere heterogeneity among plaque cell populations and aid in identifying senescent VSMCs (97). These approaches remain in methodological optimization, however, and their throughput, cost-effectiveness, and standardization fall short of large-scale clinical application. In the future, combining telomere length measurements with telomere-related protein expression and SASP markers may yield a multidimensional, high-precision system for VSMC senescence identification, providing firmer molecular evidence for risk stratification and individualized treatment of diabetic atherosclerosis (40).

## 4 Transcriptomic signatures and signaling pathway hallmarks of senescent VSMCs

### 4.1 Transcriptional regulatory network of the senescence-associated secretory phenotype

Transcriptomic analyses show that senescent VSMCs in the diabetic atherosclerotic microenvironment carry a tightly coordinated pro-inflammatory SASP gene profile, with NF-κB and MAPK signaling acting as the central regulatory hubs (12). NF-κB, the master transcription factor of SASP (98), drives transcriptional upregulation of effectors including IL-6 (99), CXCL8 (IL-8) (100), and MMP3 (101), which together build the molecular foundation of the inflammatory milieu. NAD+ metabolism feeds into this system through the NAMPT-HMGA axis, which modulates p38 MAPK and NF-κB activity and thereby sets the intensity of pro-inflammatory SASP, evidence that metabolic state and transcriptional control are closely intertwined (102). Under diabetic conditions, high glucose enhances NF-κB nuclear translocation (103), while sustained p38 MAPK activation amplifies SASP gene transcription (104). SASP gene expression is not simply linear; it is sustained by multiple positive feedback loops in which secreted factors such as IL-6 and IL-8 reactivate NF-κB in an autocrine manner, closing a self-reinforcing inflammatory cycle (105). The cGAS-STING pathway adds another layer: acting as a DNA damage sensor, it detects cytoplasmic chromatin fragments in senescent cells and activates NF-κB and STAT3, promoting SASP factor expression and secretion (106, 107). This multilayered network ensures that SASP persists and amplifies within diabetic atherosclerotic plaques (10, 12).

Epigenetic control is central to maintaining SASP persistence (108). In senescent cells, chromatin accessibility at SASP gene promoters is fine-tuned by histone-modifying enzymes (109), lending the senescent state long-term stability. SIRT1, an NAD+-dependent deacetylase, restrains IL-6 and IL-8 transcription by deacetylating H3K9 and H4K16 at SASP gene promoters; during senescence SIRT1 leaves these promoters, histone acetylation rises, and SASP gene expression climbs (109). Histone demethylases such as JMJD3 demethylate H3K27me3, a repressive mark at SASP-associated gene loci, favoring an open chromatin conformation (110). DNA methyltransferases (DNMTs) are likewise implicated in senescence-associated epigenetic reprogramming, with promoter CpG methylation among the mechanisms proposed to modulate SASP gene expression (111). Recent work in senescent human fibroblasts shows that the H2A.Z nucleosome-destabilizing mutant R80C suppresses the induction of pro-inflammatory SASP genes, an effect linked to reduced H3K27ac at SASP gene loci (112). At the post-transcriptional level, the RNA-binding protein NF90 keeps SASP factor expression low in non-senescent cells through translational repression; its decline during senescence lifts this brake and permits abundant SASP factor production (113). hnRNP A1, in turn, stabilizes SIRT1 mRNA, indirectly governing NF-κB acetylation and transcriptional activity and hence SASP output (114). Working in concert, these mechanisms ensure long-term SASP maintenance and functional output in senescent VSMCs.

A “master switch” model has recently been proposed for SASP transcriptional regulation, in which the transcription factors GATA4 and C/EBPβ cooperatively activate SASP gene expression in senescent cells, an attractive target set for multi-target interventions. In normal cells GATA4 is constitutively degraded via autophagy; in senescent cells the DNA damage response activates ATM/ATR kinases, which phosphorylate and stabilize GATA4, enabling it to cooperate with NF-κB in driving SASP gene transcription (105). C/EBPβ, another key transcription factor, assembles with NF-κB into an enhanceosome complex at SASP gene promoters and promotes the expression of IL-6, IL-8, and related factors. IRF1 (interferon regulatory factor 1) has also been found to bind the promoter of TNFSF13B (encoding BAFF) during senescence and stimulate its transcription, with BAFF feeding back on the senescence phenotype through NF-κB and p53 pathways (115). The NLRP1 inflammasome, acting as a cGAS-dependent DNA damage sensor, mediates SASP factor release through GSDMD and further expands the SASP regulatory network (116). These “master switch” transcription factors deepen our mechanistic understanding of SASP regulation and lay a theoretical foundation for multi-target intervention in diabetic atherosclerosis: simultaneously targeting GATA4, C/EBPβ, and NF-κB may suppress excessive SASP activation more effectively and delay atherosclerotic progression (117).

### 4.2 Cell cycle gene expression modules and senescence transcriptomic signatures

Cell cycle-related gene modules in senescent VSMCs show a pronounced transcriptional silencing signature (88, 118). Using systems biology approaches such as weighted gene co-expression network analysis (WGCNA), integrated transcriptomic analyses have linked co-expression gene modules and machine-learning candidate regulators to senescence-related pathways under diabetic conditions (118), alongside the established shutdown of E2F target genes and proliferation checkpoints during cellular senescence (88). This transcriptional change underlies stable cell cycle arrest, with activation of the p53-p21 and p16-Rb pathways at its core (119). In a VSMC senescence model of diabetic kidney disease (DKD), for example, integrated bioinformatics and machine learning identified MMP14 and FOS as key regulatory genes that control senescence by perturbing cell cycle distribution (e.g., inducing G0/G1 arrest) (118). High glucose can likewise upregulate TXNIP, suppress AKT phosphorylation, activate p53, and ultimately drive VSMC senescence and atherosclerotic progression (38). Dysregulation of cell cycle gene expression modules thus stands as a core transcriptional feature of VSMC senescence, with consequences extending beyond proliferative arrest to shape cellular secretory phenotype and function (10, 12).

Non-coding RNAs, microRNAs (miRNAs) and long non-coding RNAs (lncRNAs) in particular, have become increasingly prominent in the senescence transcriptional regulatory network. Downregulation of the miR-29 family in senescent VSMCs derepresses targets such as type I collagen α1 chain (COL1A1), contributing to senescence-associated fibrosis and matrix remodeling (119). miR-34a, by contrast, is upregulated with age in the aorta, where it induces VSMC senescence and promotes vascular calcification through direct downregulation of SIRT1 (62). lncRNAs matter too: lncRNA-ES3 is upregulated in high-glucose-induced VSMC calcification/senescence and, acting as a competitive endogenous RNA (ceRNA), sponges miR-34c-5p to relieve inhibition of the target gene BMF and favor the senescence phenotype (32). A further study implicated circRNA-0077930 in intercellular communication, showing that exosomes from high-glucose-stimulated endothelial cells deliver this circRNA to VSMCs and induce senescence via the miR-622/Kras axis (65). Together these findings paint a complex non-coding RNA regulatory network that fine-tunes senescence-associated gene expression through ceRNA mechanisms and epigenetic regulation.

Transcriptional repression of telomerase reverse transcriptase (TERT) marks cellular senescence. As VSMCs senesce, progressive telomere shortening and dysfunction drive replicative senescence (120). TERT, the catalytic subunit of telomerase, is downregulated, directly lowering telomerase activity and accelerating telomere attrition, which triggers DNA damage responses and cell cycle arrest (26). In diabetic atherosclerotic models, oxidative stress damages VSMC telomeres, whereas agents such as rutin protect telomeres and suppress premature VSMC senescence (26). RNAscope *in situ* hybridization offers a powerful spatial transcriptomic tool for visualizing TERT expression at single-cell resolution in tissue sections: it detects TERT mRNA molecules within individual cells with high sensitivity and specificity and reveals their spatial distribution in senescent plaques. Combined with conventional immunohistochemistry and transcriptomic sequencing, RNAscope links TERT expression to cellular morphology, histopathological features, and other senescence markers (e.g., p21 and SA-β-gal), markedly enhancing the spatial resolution of transcriptomic analysis (58). Such multidimensional strategies help localize senescent cell “hotspots” within the vascular wall and clarify their heterogeneity in the diabetic atherosclerotic microenvironment.

### 4.3 Single-cell sequencing reveals the heterogeneity of senescent VSMCs

scRNA-seq has opened a powerful window onto the heterogeneity of senescent VSMCs in diabetic atherosclerotic plaques at single-cell resolution. Multiple studies have identified several VSMC subpopulations in human and mouse plaques, with “senescent-like” and “osteoblast-like” subsets attracting particular attention because of their tight association with disease progression. The transcriptional profiles of these subsets partially overlap with those of classical *in vitro* senescence models (replicative senescence or stress-induced premature senescence) but also diverge substantially, indicating that the *in vivo* microenvironment shapes the VSMC senescence phenotype (64, 67, 121).

Trajectory inference suggests that senescent VSMCs arise through a continuous state transition from a contractile to a synthetic and then to a senescent phenotype. Along this path, marker genes such as Mmp3, Serpine1, and Igfbp3 change expression sequentially (122). These genes participate in extracellular matrix degradation and remodeling and are closely tied to SASP establishment (120). Serpine1 (encoding plasminogen activator inhibitor-1, PAI-1) has been confirmed as a key senescence marker by multiple studies: PAI-1 acts as an essential mediator of cellular senescence (123), and SERPINE1 is co-induced with p53 in ox-LDL-exposed aortic endothelial cells and macrophages undergoing senescence (124). PAI-1 upregulation in senescent VSMCs may shift the local proteolytic balance toward matrix deposition and vascular fibrosis (125). Senescent VSMCs release active matrix metalloproteinases and secrete less collagen (7); rising Mmp3 may likewise destabilize the vascular wall by degrading connective tissue matrix components such as collagen and proteoglycans, favoring plaque instability (126–128). In diabetic atherosclerosis, plaques accumulate more senescent VSMCs and fewer smooth muscle cells, and inhibition of premature VSMC senescence attenuates lesion burden and stabilizes the plaque (26). This contractile-to-senescent transition, marked by lost proliferative capacity, heightened SASP factor secretion, and a drift toward osteoblast-like differentiation, ultimately culminates in vascular calcification and arteriosclerosis (51, 129). The senescent phenotype thereby becomes a driver of plaque vulnerability: senescent VSMCs release matrix metalloproteinases that degrade plaque connective tissue (7, 126), and diabetes-associated VSMC senescence promotes osteogenic differentiation and calcification (51).

A related question is whether senescent VSMCs represent a distinct cellular state or merely a stage within the continuum of VSMC phenotypic modulation. The two processes share transcriptional features—downregulation of contractile genes such as Acta2 and Myh11, upregulation of synthetic and inflammatory genes, and a shift toward osteogenic programs (16, 129)—but they are distinguished by one defining feature: irreversible growth arrest. Reversible phenotypic switching between contractile and synthetic states allows VSMCs to resume proliferation and migration in response to environmental cues, whereas senescent VSMCs cannot re-enter the cell cycle even under strong mitogenic stimulation, owing to stable p16-Rb and p53-p21 pathway activation (5, 6). Trajectory inference from single-cell data suggests that senescence arises as a late branch of the phenotypic modulation continuum rather than as an entirely separate lineage (16), and senescent VSMCs co-opt many of the same synthetic and osteogenic transcriptional programs (16, 129). Nevertheless, senescence carries additional, non-reversible hallmarks—p16/p21 upregulation, SA-β-gal activity, a robust SASP, and chromatin remodeling that locks the growth-arrested state—that are absent from reversible phenotypic switching (5, 6). A practical operational distinction can therefore be drawn: a VSMC can be classified as senescent only when it displays both phenotypic modulation features and irreversible growth arrest confirmed by independent senescence markers plus failure of mitogenic re-stimulation (6, 16). This two-tier definition would help make single-cell and trajectory studies more comparable and would reduce the risk of conflating transient phenotypic modulation with true senescence.

Cross-study comparisons, however, show that identifying senescent VSMCs in scRNA-seq data is heavily dependent on the consistency of cluster markers across laboratories. The overlap among gene sets used to define senescent VSMCs is currently limited, complicating data integration and comparison. A unified consensus signature should integrate key genes spanning cell cycle inhibitors (e.g., Cdkn2a/p16, Tp53/p53), SASP factors (e.g., IL-6, IL-1β, MMP3), and osteogenic differentiation markers (e.g., RUNX2, ALPL) (12, 122). Cdkn2a, a classical senescence marker, has been used to label senescent VSMCs in multiple studies, yet its expression can differ markedly among subpopulations (16). The Tp53 pathway also occupies a central regulatory position in VSMC senescence, with downstream targets such as p21 causing cell cycle arrest and mitochondrial dysfunction (57). Establishing a consensus gene set that incorporates multidimensional markers is therefore essential for improving the accuracy and reproducibility of senescent VSMC identification.

Spatial transcriptomics has recently begun to reveal where senescent VSMCs sit within plaques. They tend to localize to the fibrous cap center and the plaque-media junction and show notable spatial proximity to calcified regions (12). This distribution suggests that senescent VSMCs concentrated in these plaque regions express a pro-inflammatory and matrix-remodeling secretory program, including Spp1, Ctsb and Tnfrsf11b (12). Senescence-associated inflammatory cytokines link senescent VSMCs to intensified vascular inflammation, with IL-6 identified as the key driver of VSMC senescence in human arterial lesions (130). The spatial proximity of senescent VSMCs to calcified regions further hints that they promote osteoblast-like differentiation of neighboring VSMCs by releasing osteoinductive factors (e.g., BMP-2) or through extracellular vesicle-mediated communication, accelerating vascular calcification (12, 57, 129). These spatial observations supply key clues about the specific mechanisms by which senescent VSMCs drive atherosclerotic plaque progression.

## 5 Metabolic reprogramming and the identification of senescent VSMCs

### 5.1 Effects of abnormal glucose metabolism on the senescent phenotype

Metabolic reprogramming is a defining feature of the VSMC senescent phenotype, most strikingly the switch from oxidative phosphorylation to aerobic glycolysis (the Warburg effect) (40). In calcifying VSMCs, the glycolytic enzyme PFKFB3 is upregulated; its knockdown reduces glycolysis and osteogenic transdifferentiation, whereas lactate supplementation reverses these effects, indicating that enhanced glycolysis drives VSMC osteogenic conversion (131). In parallel, calcifying and senescent VSMCs show altered mitochondrial dynamics-elongated mitochondria, elevated mitochondrial ROS and reduced mitophagy-changes tied to the senescence program (57). Metabolic reprogramming under high-glucose conditions does more than sustain synthetic-phenotype activities: through the lactate/GPR81 pathway it reinforces the synthetic VSMC phenotype (63), and glycolytic shifts such as PFKFB3-driven lactate production are likewise coupled to osteogenic, pro-calcifying VSMC states (131). Cathepsin D (CTSD) inhibits AGE-induced VSMC phenotypic conversion by regulating the glycolytic pathway, underscoring how metabolic enzymes govern the reprogramming of senescent VSMCs (54). Accumulated glycolytic intermediates further feedback on energy-sensing pathways such as AMPK and mTORC1, influencing the maintenance of growth arrest (40). This metabolism-senescence interplay constitutes a molecular basis of the VSMC senescent phenotype in diabetic vascular disease.

Sustained hyperglycemia strengthens the hexosamine biosynthetic pathway (HBP) and the polyol pathway, and this altered metabolic flux figures prominently in the metabolic reprogramming of senescent VSMCs. High glucose drives reprogramming by enhancing AGE formation and accumulation, activating diacylglycerol-dependent protein kinase C (PKC), and disrupting pentose phosphate pathway (PPP) flux and HBP homeostasis (132). These pathway changes affect not only cellular energy metabolism but also senescence signal transduction, intensifying oxidative stress, inflammatory cascades, and the imbalance between autophagy and apoptosis (132). Under high-glucose conditions, ChREBP, the upstream transcriptional activator of TXNIP, raises TXNIP expression and induces VSMC senescence by suppressing AKT phosphorylation; senescence in turn activates p53 and increases Mig6 expression, which negatively regulates epidermal growth factor receptor activity and furthers atherosclerotic progression, together establishing the ChREBP/TXNIP/AKT axis as a distinct route by which hyperglycemia drives VSMC senescence in diabetic macrovascular complications (38). High glucose and insulin resistance also act synergistically to accelerate atherosclerosis through AGE accumulation, enhanced oxidative stress, NLRP3 inflammasome activation, and VSMC phenotypic switching (133). The interplay between these metabolic flux changes and senescence signal activation forms a metabolism-senescence vicious cycle, rendering VSMCs in diabetes more prone to entering the senescent phenotype and sustaining SASP secretion (40, 134).

Metabolomic studies have further exposed how glycolytic intermediate accumulation shapes the VSMC senescent state. In senescent VSMCs, accumulation of glycolytic intermediates reflects enhanced glycolytic flux (131), and concomitant changes in the AMPK/TSC2/mTORC1 axis-declined AMPK/TSC2 phosphorylation with elevated mTORC1/S6K1 phosphorylation-accompany replicative senescence and its stable growth arrest; pharmacological activation of AMPK or inhibition of mTORC1 delays this senescence program (135). AMPK, a cellular energy sensor activated under energy stress, regulates metabolism and autophagy by phosphorylating downstream substrates, whereas mTORC1, a key controller of cell growth and proliferation, responds to nutrient and growth factor signals (135). SIRT1, an NAD+-dependent deacetylase, is downregulated in aged VSMCs; its reduction increases p21-dependent vascular cell senescence and enhances NF-κB-driven inflammatory signaling in the vessel wall, whereas VSMC-specific SIRT1 overexpression suppresses both (136). Reciprocally, sustained mTORC1 activation accompanies VSMC replicative senescence, and mTORC1 inhibition delays it (135). Accumulated glycolytic intermediates under high-glucose conditions can influence autophagic levels and protein synthesis rates through the AMPK-mTORC1 axis, thereby regulating maintenance of the senescence-associated phenotype (137). Epigenetic regulation also interacts with metabolic reprogramming in senescent VSMCs. Several miRNAs are aberrantly expressed in diabetes: miR-504 rises significantly in diabetic VSMCs and promotes dysfunction and inflammatory gene expression by targeting Grb10 and Egr2; these changes persist even after *in vitro* culture, implicating them in the metabolic memory of vascular complications (46). Similarly, miR-145 remains persistently elevated in VSMCs of patients with type 2 diabetes, coexisting with DNA damage and senescence features; it promotes senescence-associated cytokine secretion by activating p38α mitogen-activated protein kinase and reinforces the senescent phenotype in neighboring cells in a paracrine manner (11). Abnormal glucose metabolism thus profoundly shapes the VSMC senescent phenotype through reprogramming of multiple metabolic pathways and accumulation of intermediate metabolites, providing a basis for understanding how diabetes accelerates vascular aging (71, 138).

### 5.2 Lipid metabolism disorders and senescence-related signaling

In diabetic atherosclerosis, lipid metabolism disorders and the VSMC senescent phenotype are closely intertwined. Uptake of oxidized low-density lipoprotein (oxLDL) is a key step in VSMC foam cell formation, and it is especially prominent in senescent VSMCs. The scavenger receptors CD36 and SR-A play central roles here; their upregulation directly promotes abnormal lipid influx and accumulation. Aberrant CD36 expression and function is a hallmark of lipid metabolism dysfunction across multiple metabolic disorder models (139). In non-alcoholic fatty liver disease models, for example, iron overload aggravates lipid disorders by upregulating CD36 and fatty acid synthase (FAS) (140). Within atherosclerotic plaques, VSMCs can acquire foam-cell-like characteristics: CD36 mediates oleic acid-induced lipid accumulation and foam cell formation in VSMCs, and SR-A overexpression likewise drives acetylated/oxidized LDL uptake and foam cell formation in aortic SMCs. Given that senescent VSMCs secrete SASP factors, CD36 and SR-A may serve as auxiliary markers for identifying the foam-cell-like state of senescent VSMCs in plaques (141, 142).

Transcriptional reprogramming of lipid metabolism genes is another core feature of senescent VSMCs. Expression of sterol regulatory element-binding protein 2 (SREBP2), fatty acid synthase (FASN), and stearoyl-CoA desaturase 1 (SCD1) changes significantly in senescent cells, reducing fatty acid oxidation and increasing lipid synthesis. This metabolic shift tracks closely with sustained SASP activation. In diabetic kidney disease research, senescence of renal tubular cells is likewise accompanied by suppressed fatty acid β-oxidation (FAO): high glucose reduces CPT1, ACOX1 and PPAR-α expression in tubular epithelial cells and promotes lipid accumulation, an effect reversible by restoring FAO (143). Dysregulation of the SREBP pathway is further considered a key driving mechanism of lipid synthesis in this setting (144). In diabetic non-alcoholic fatty liver disease models, rutin improves lipid metabolism by activating the AMPK/SREBP1 pathway, further confirming the centrality of SREBP signaling in lipid homeostasis (145). In senescent VSMCs, sustained SREBP2 activation promotes cholesterol and fatty acid synthesis and aggravates intracellular lipid accumulation by upregulating FASN and SCD1, providing a metabolic foundation for persistent SASP activation.

The sphingomyelin-ceramide pathway is central to lipotoxic stress in senescent VSMCs. Ceramide, an important bioactive sphingolipid, enhances senescence signal transduction by activating the p38/JNK stress signaling pathway as it accumulates. In Sertoli cell senescence, lipid droplet-driven ROS production can trigger ribosome collisions that activate the ZAKα-p38 signaling axis and lead to cellular senescence (146), revealing a direct link between lipid metabolism disorders and senescence signaling. In VSMCs, ceramide accumulation likewise promotes an inflammatory phenotype: in human plaques, ceramide and related sphingolipids are elevated and correlate with inflammatory cytokines and histological markers of plaque instability, and *in vitro* ceramide induces inflammatory responses in human coronary VSMCs (147). In liver aging research, miR-23b-3p delivered via small extracellular vesicles promotes cellular senescence and aberrant lipid metabolism, further suggesting a complex regulatory network between lipid metabolism and senescence signaling (148). Ceramide and its downstream p38/JNK pathway may therefore constitute a metabolic-level marker combination for identifying and intervening in the pathological process of senescent VSMCs in diabetic atherosclerosis.

## 6 Critical appraisal and comparison of methodological validity

The multidimensional approaches currently used to identify senescent VSMCs, along with their strengths and limitations, are compared in **Figure 2**.

**Figure 2:**
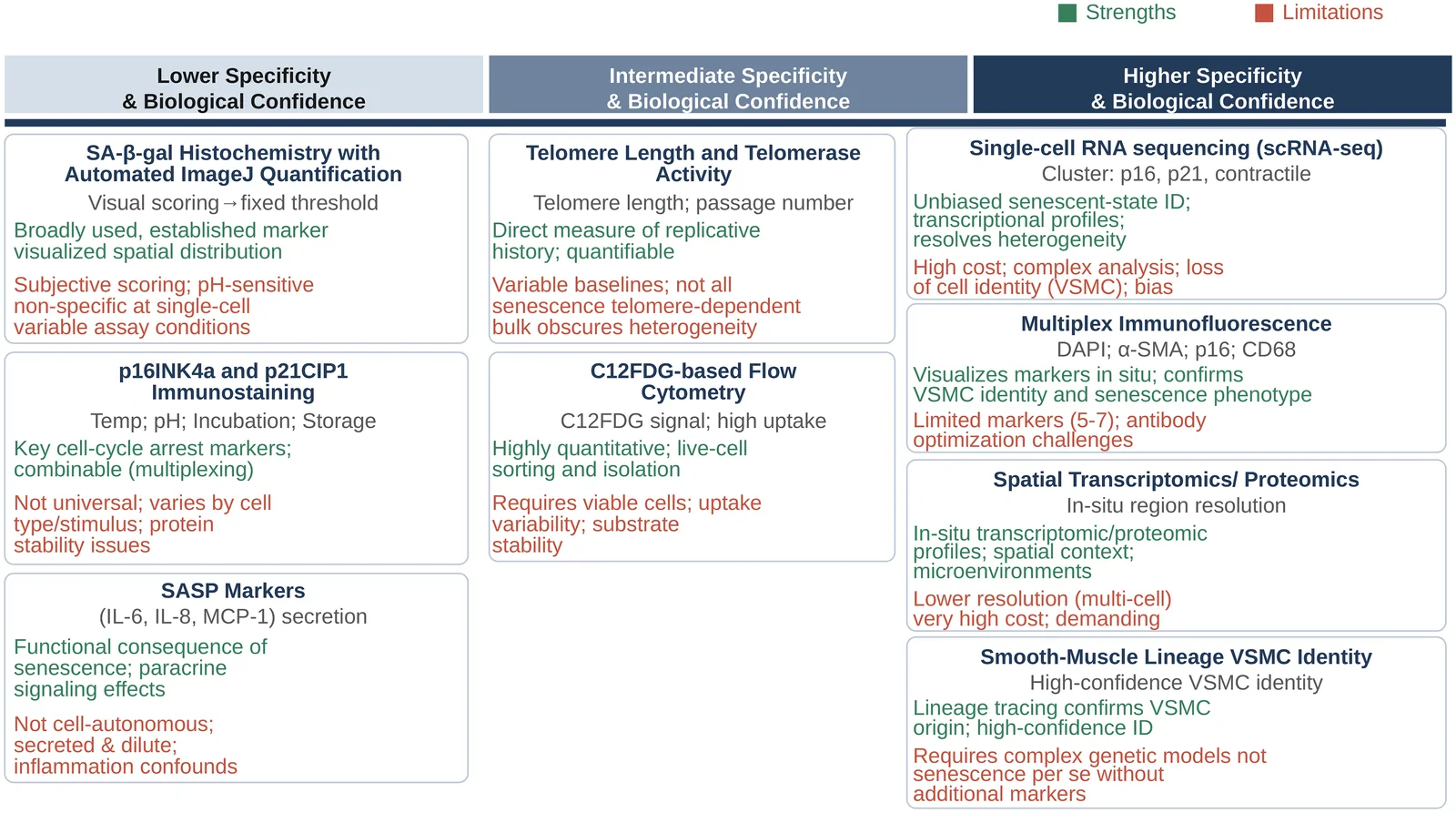
Multidimensional approaches for identifying senescent VSMCs and their methodological strengths and limitations. Detection methods are arranged from lower to higher specificity and biological confidence, including SA-β-gal histochemistry with automated ImageJ quantification, p16INK4a and p21CIP1 immunostaining, SASP marker detection, telomere length and telomerase activity assays, C12FDG-based flow cytometry, single-cell RNA sequencing (scRNA-seq), and multiplex immunofluorescence. The methodological gap between *in vitro* models (high-glucose or H2O2-induced VSMCs) and *in vivo* plaques is highlighted, together with smooth-muscle lineage tracing (Myh11-CreERT2) and spatial transcriptomics/proteomics. H2O2, hydrogen peroxide; IL, interleukin; MCP-1, monocyte chemoattractant protein-1; DAPI, 4′,6-diamidino-2-phenylindole; α-SMA, α-smooth muscle actin; scRNA-seq, single-cell RNA sequencing. In each method panel, strengths (green text) are listed above limitations (red text), so that the two categories can be distinguished by position as well as by color.

### 6.1 Comparative analysis of sensitivity and specificity of senescence detection methods

No single marker yet achieves highly specific detection of senescent VSMCs, and this gap is especially acute in diabetic atherosclerosis research. p16Ink4a, a cyclin-dependent kinase inhibitor, is a widely used senescence marker; however, its expression is not universal and may be absent or delayed in some stress-induced senescence models. SA-β-gal detection, by contrast, is widely used for its simplicity and low cost; however, false positives can arise because confluent quiescent cells, serum starvation, and oxidative stress all increase SA-β-gal activity independently of senescence (149). Combining p16Ink4a with SA-β-gal and additional markers is therefore preferable, yet specificity remains vulnerable to the inflammatory microenvironment and metabolic stress. Under diabetic conditions, high glucose itself can induce stress-induced premature senescence of VSMCs while activating inflammatory signaling, producing non-specific senescence marker expression (9). The p53-p21 pathway, another important senescence effector, is central to oxidative stress- and DNA damage-induced VSMC senescence, but its expression is also governed by multiple pathophysiological factors, complicating single-marker interpretation (57, 150).

On the technical side, combining flow cytometry with single-cell transcriptomics offers a fresh route to precise senescent VSMC identification. Flow cytometric SA-β-gal detection using the C12FDG substrate enables quantitative analysis and subpopulation sorting of senescent cells, and in diabetic plaque models its results correlate well with single-cell transcriptomic data, hinting that this combined strategy may mature into a next-generation standardized detection route (130). scRNA-seq resolves VSMC heterogeneity at single-cell level and pinpoints senescence-specific transcriptional features: upregulation of cell cycle inhibitory genes, heightened SASP factor expression, and activation of pro-calcification genes (129, 151). Yet the transcriptomic signatures obtained from *in vitro* high-glucose or H2O2-induced senescence models show only moderate concordance with the true transcriptional state of plaque VSMCs *in vivo*. The discrepancy likely reflects the inability of *in vitro* culture to recapitulate the complex *in vivo* microenvironment, including mechanical signals from the extracellular matrix, paracrine effects of immune cells, and local hypoxia (6, 120). *In vivo* senescent VSMCs are also often accompanied by mitochondrial dysfunction, impaired autophagic flux, and phenotypic switching, features that simple *in vitro* stress models reproduce only partially (152, 153). When interpreting senescence marker data derived from *in vitro* models, the impact of methodological differences on inference accuracy must therefore be weighed carefully, and multidimensional detection approaches combined (118, 154).

### 6.2 Necessity and challenges of multi-marker combination strategies

Identifying senescent VSMCs is a pivotal step in diabetic atherosclerosis research, yet no single biomarker fully captures the complex biological features of these cells. We recommend combining at least one cell cycle inhibitor (p16 or p21), one functional indicator (SA-β-gal activity or loss of proliferation), and one metabolic indicator (mitochondrial function or glycolytic activity) for specific senescent VSMC identification. The rationale lies in the multidimensional nature of senescence. Cell cycle inhibitors reflect the core molecular mechanism of senescence (cell cycle arrest), with p16 and p21 mediating irreversible growth arrest through the p16-Rb and p53-p21 pathways, respectively (119). SA-β-gal, the most classical functional senescence marker, reflects lysosomal β-galactosidase activity accumulated in senescent cells and has been used extensively to detect VSMC senescence under diabetic conditions (1, 155). Metabolic indicators capture the reprogramming of energy metabolism in senescent cells; VSMCs shift from oxidative phosphorylation toward glycolysis during senescence, with mitochondrial dysfunction and enhanced glycolytic activity as prominent features (156). Under diabetic high-glucose conditions, CTSD regulates AGE-induced VSMC phenotypic conversion by suppressing the glycolytic pathway, underscoring the irreplaceable value of metabolic indicators in senescence identification (54). Multiple studies also confirm that p16 and p21 are coordinately upregulated in diabetes-induced VSMC senescence: high glucose simultaneously raises p21, p53, and p16 through the circRNA-0077930-miR-622-Kras ceRNA network (65), and the combined action of angiotensin II and high glucose promotes VSMC senescence through the p21-pRb pathway (157). Combining these three categories of indicators thus addresses senescence from the molecular mechanism, phenotypic features, and metabolic state dimensions, substantially improving specificity and accuracy.

Building on the multi-marker strategy outlined above, we propose a stepwise validation framework for defining senescent VSMCs in diabetic atherosclerosis, which we recommend as a practical consensus criterion for the field. In the first step, VSMC identity must be confirmed—either by lineage tracing (e.g., Myh11-CreERT2) or by co-expression of contractile markers such as MYH11, ACTA2, and TAGLN, combined with exclusion of macrophage and endothelial lineage markers. In the second step, stable cell-cycle arrest is assessed: upregulation of p16INK4a and/or p21CIP1, hypophosphorylated Rb, and absence of Ki-67 and PCNA together provide molecular evidence of growth arrest. In the third step, functional senescence features are verified: SA-β-gal activity at pH 6.0, DNA damage foci (γ-H2AX or 53BP1), and a sustained SASP profile (e.g., IL-6, CXCL8, MMP3) provide evidence that the arrested state is biologically active rather than quiescent. In the fourth step, lineage persistence is confirmed: the cell must retain VSMC lineage markers despite losing contractile function, distinguishing senescent VSMCs from transdifferentiated cell types such as macrophage-like or osteoblast-like cells that may themselves express senescence markers. We consider that a cell meeting criteria at all four steps can be classified as a “high-confidence senescent VSMC”, whereas cells meeting only some criteria should be described as “senescent-like” to avoid overclaiming. This four-step framework is consistent with the multi-marker, lineage-aware strategy summarized in **Figure 3**, but we present it here explicitly as a proposed consensus standard to guide future studies and cross-study comparison.

**Figure 3:**
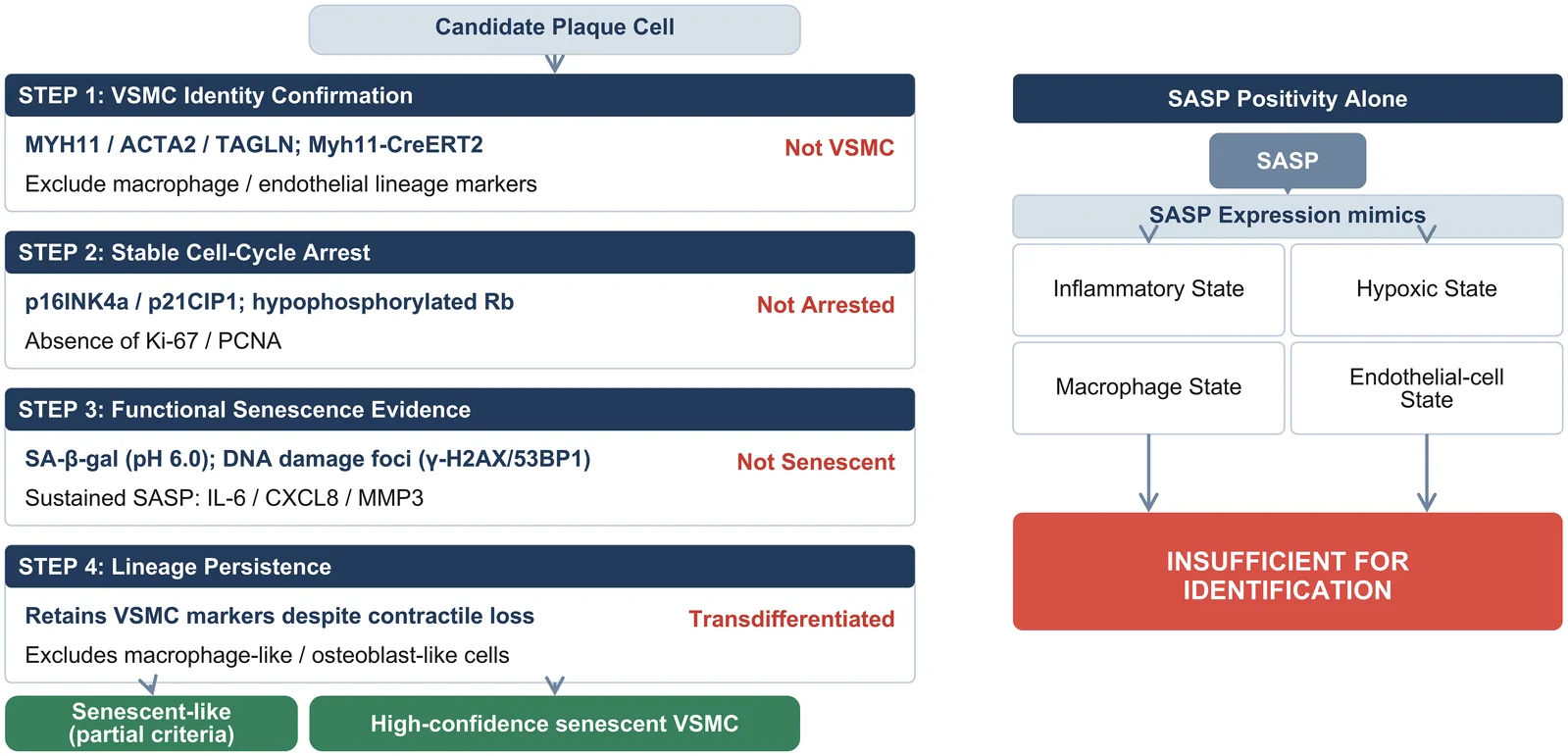
A multi-marker and lineage-aware four-step strategy for high-confidence identification of senescent VSMCs. A candidate plaque cell is sequentially assessed through four steps for VSMC identity (MYH11, ACTA2, TAGLN, and Myh11-CreERT2 lineage tracing, with exclusion of macrophage and endothelial lineage markers), stable cell-cycle arrest (p16INK4a, p21CIP1, hypophosphorylated Rb, and absence of Ki-67 and PCNA), functional senescence evidence (SA-β-gal activity at pH 6.0, DNA damage foci such as γ-H2AX or 53BP1, and a sustained SASP such as IL-6, CXCL8, and MMP3), and lineage persistence (retention of VSMC lineage markers despite contractile loss, excluding transdifferentiated macrophage-like and osteoblast-like cells). A cell meeting all four criteria is classified as a high-confidence senescent VSMC, whereas a cell meeting only some criteria is described as senescent-like. SASP positivity alone is insufficient for identification because it can mimic inflammatory, hypoxic, macrophage, and endothelial-cell states. 53BP1, p53-binding protein 1; ACTA2, α-smooth muscle actin; CXCL8, C-X-C motif chemokine ligand 8; γ-H2AX, phosphorylated histone H2AX; IL-6, interleukin-6; MMP3, matrix metalloproteinase 3; MYH11, smooth muscle myosin heavy chain; PCNA, proliferating cell nuclear antigen; Rb, retinoblastoma protein; SA-β-gal, senescence-associated β-galactosidase; SASP, senescence-associated secretory phenotype; TAGLN, transgelin.

Multi-marker co-localization in tissue sections can be achieved with multiplex immunofluorescence staining; the OPAL system visualizes more than six markers simultaneously and provides whole-slide scanning with automated quantitative analysis, markedly boosting throughput and objectivity. This platform permits precise evaluation of the localization and density of senescent VSMCs in atherosclerotic plaques. In human atherosclerotic plaques, VSMCs within the fibrous cap express senescence markers including SA-β-gal, p16, and p21, which are absent in normal vessels, providing direct *in situ* evidence that VSMC senescence accompanies plaque progression (4, 158). Even so, multi-marker combinations that substantially reduce false-positive rates can still be misled by SASP-like expression profiles of non-senescent cells in inflammatory and hypoxic plaque regions. SASP factors secreted by senescent cells (pro-inflammatory cytokines, chemokines, and matrix metalloproteinases) can also be expressed by non-senescent cells in these regions, interfering with accurate senescent cell identification (61). The chronic inflammatory state of diabetes itself can induce VSMC senescence; high glucose and angiotensin II act synergistically to promote VSMC senescence through oxidative stress and autophagy (157), making marker interpretation in inflammatory regions more complex. To confirm cellular origin, lineage tracing technologies such as the Myh11-CreERT2 mouse model, which specifically labels smooth muscle cells and their progeny, should be applied to track the origin and fate of senescent VSMCs at tissue level. Combining lineage tracing with multi-marker staining confirms the smooth muscle origin of senescent cells at single-cell resolution, effectively excluding macrophages, endothelial cells, or other cell types, and offers a more reliable methodological basis for senescence-targeted therapy.

A multi-marker, lineage-aware decision strategy for the high-confidence identification of senescent VSMCs is summarized in **Figure 3**.

### 6.3 Impact of spatial heterogeneity on detection results and coping strategies

Atherosclerotic plaques are far from homogeneous; different regions (fibrous cap, shoulder regions, necrotic core periphery) differ markedly in oxygen tension, nutrient supply, and inflammatory factor concentrations. This spatial heterogeneity of the microenvironment profoundly shapes the expression and detection of senescence markers in VSMCs. In diabetic atherosclerotic plaques, for instance, necrotic core peripheral regions may drive VSMC apoptosis or senescence through hypoxia and nutrient deprivation, whereas fibrous cap regions may develop distinct senescent phenotypes under the combined influence of mechanical stress and inflammatory factors. Insulin resistance (IR) aggravates plaque instability by reducing VSMC survival and upregulating the CX3CL1/CX3CR1 axis, with intensity that may vary across plaque regions (55). Hyperglycemia likewise induces VSMC senescence through the ChREBP/TXNIP/AKT axis, whose activation may be spatially non-uniform depending on local glucose concentrations and oxidative stress levels (38). Homogenate analysis of whole plaques can therefore mask region-specific molecular alterations, leading to underestimation or misjudgment of overall senescence marker detection rates.

Laser capture microdissection (LCM) offers a high-precision solution to spatial heterogeneity. This technique isolates VSMCs from specific regions (e.g., fibrous cap or shoulder) of tissue sections for downstream transcriptomic or proteomic analysis, substantially sharpening the targeting of senescence marker detection. In early studies, LCM combined with quantitative PCR successfully analyzed the distribution of non-esterified fatty acids (NEFA) in different regions of atherosclerotic plaques from diabetic and non-diabetic patients and its relationship with local inflammation, confirming the strong influence of the local metabolic microenvironment on plaque biological properties (159). The principal challenge of LCM, however, is RNA degradation during tissue processing: tissue fixation, staining, laser cutting, and collection involve multiple steps in which RNA is highly vulnerable (160). Stringent quality control, for example ensuring an RNA integrity number (RIN) greater than 7, is therefore essential to guarantee the reliability and reproducibility of downstream sequencing or qPCR data.

Beyond LCM, co-registration of spatial transcriptomics with multiplex fluorescence immunohistochemistry (mIHC) on serial sections is emerging as an effective approach that preserves morphological information while capturing high-dimensional molecular features. This method maps gene expression data directly onto histological structures, resolving the microenvironmental distribution of VSMC senescence in the spatial dimension. Combining mIHC-labeled cell types (e.g., senescent VSMCs and macrophages) with spatial transcriptomic data can reveal the transcriptomic signatures of VSMCs in different plaque regions and their interaction networks with surrounding immune cells (12). Studies indicate that diabetes increases arterial miR-221/222 expression, which down-regulates p27Kip1 in VSMCs and exacerbates intimal thickening following vascular injury (161). Given the complex role of Wnt signaling in coronary artery disease, particularly its contribution to vascular calcification (162), spatial analysis methods may help clarify how this pathway regulates VSMC phenotype across plaque regions. This multimodal integration strategy provides a powerful tool for understanding intra-plaque cellular senescence heterogeneity and its mechanisms in diabetic atherosclerosis progression.

## 7 Clinical translation prospects of senescent VSMCs in diabetic atherosclerosis

### 7.1 Potential of senescence markers for predicting plaque vulnerability

The transition of atherosclerotic plaques from stable to vulnerable phenotypes underlies acute cardiovascular and cerebrovascular events, and VSMC senescence with its accompanying SASP contributes centrally to this process (163). As circulating biological mediators, SASP factors offer a non-invasive window onto the senescent cell burden within plaques. IL-6, a core SASP component, is highly expressed in senescent VSMCs and released into the extracellular space; its serum levels correlate significantly and positively with carotid plaque instability and decline dynamically after stroke, suggesting that it may reflect senescence-associated inflammatory activity inside plaques (164). MMP-9, a key extracellular matrix-degrading enzyme, weakens the collagen framework of the fibrous cap when elevated; circulating MMP-9 levels track closely with plaque vulnerability, including thinner fibrous caps and intraplaque hemorrhage (165) (166). Pentraxin 3 (PTX3), a sensitive marker of local inflammation, shows a significant negative correlation between its coronary sinus plasma concentration and plaque CT density, and its value for indicating plaque vulnerability exceeds that of high-sensitivity C-reactive protein (167). Myeloid-derived markers such as monocyte chemoattractant protein-1 (MCP-1) and myeloperoxidase (MPO) also rise markedly in the acute phase of plaque destabilization and can distinguish stable from unstable plaques (168, 169). Individual SASP factors, however, have limited specificity and sensitivity, and a 76-protein multi-marker panel has been proposed as a prognostic signature of plaque vulnerability, an approach more consistent with the complexity of plaque destabilization (170). The apolipoprotein B/A1 ratio has been confirmed as an independent predictor of plaque rupture, erosion, and thrombosis, outperforming LDL cholesterol (171). Although circulating SASP factors hold promise as “liquid biopsy” indicators, most existing studies are cross-sectional or small-sample cohorts; large-scale prospective studies are still needed to validate their independent predictive value and clinical cutoff standards (172).

Positron emission tomography (PET) combined with emerging tracers offers new technical routes for non-invasive vascular imaging. 18F-fluorodeoxyglucose (18F-FDG), the classical inflammation imaging agent, provides a noninvasive measure of plaque inflammation in patients, correlating with macrophage infiltration within carotid plaques (173); it does not, however, specifically reflect the senescent cell burden. Whereas senescence-specific PET tracers remain largely investigational, probes responsive to senescence-associated β-galactosidase (SA-β-gal) have recently enabled noninvasive imaging of plaque senescence in the aortic wall of mice, with signal trends reflecting vascular smooth muscle cell senescence (174). Beyond cellular senescence itself, structural vulnerability can also be probed noninvasively: 18F-sodium fluoride (18F-NaF) PET/computed tomography (CT), which detects active microcalcification, was shown in a prospective clinical trial to identify and localize ruptured and high-risk coronary atherosclerotic plaques (175). High-resolution micro-computed tomography (micro-CT) of human coronary fibrous caps further demonstrates that clustered microcalcifications ≥5 μm enhance local tissue stress, with a steep increase when the gap-to-diameter ratio (h/D) is below 0.4 and particles are aligned along the tensile axis of the cap; only a small subset of caps thus has the potential for rupture (176). Even at the cellular scale, microcalcifications of ~10 μm in diameter concentrate local stress and drive interfacial debonding in thin fibrous caps (177). Because 18F-NaF PET/CT enables noninvasive detection and localization of such ruptured and high-risk plaques *in vivo*, it can inform risk stratification in patients with coronary artery disease (175).

These integrated imaging and molecular marker strategies are moving the noninvasive detection of senescent VSMCs from preclinical research toward translational applications (178), but validation of their specificity for senescent VSMCs in diabetic patients and the establishment of standardized imaging protocols remain major challenges (179).

Correlation studies between imaging indicators and vascular wall senescence markers are still in their early stages, yet the potential of imaging surrogates has drawn considerable attention. The coronary artery calcium (CAC) score, a widely used clinical imaging indicator, does not bear a simple linear relationship to the burden of senescent VSMCs within plaques. Calcium content in unstable plaques is significantly lower than in stable plaques, whereas the spectral curve slope shows the opposite pattern, suggesting that the pattern of plaque calcification (microcalcification vs. macrocalcification), rather than total calcium content, determines plaque vulnerability (180). Gemstone spectral imaging (GSI), an emerging CT technology, quantifies intra-plaque calcium content and spectral curve slope; unstable carotid plaques show significantly reduced calcium content and elevated spectral curve slopes, echoing changes in serum Hs-CRP and MCP-1 and supporting combined imaging-serum multimodal assessment of plaque vulnerability (180). Spatial proteomic studies have further revealed differences in molecular programs between plaque regions (necrotic core, fibrous cap): proteins such as PCSK9 are significantly overexpressed in thin-cap fibroatheromas, and *in vitro* experiments confirm that oxidative stress and inflammatory stimulation promote PCSK9 secretion by primary VSMCs, providing direct evidence linking imaging phenotypes to molecular mechanisms (181). Optical coherence tomography (OCT) identifies vulnerable features such as thin-cap fibroatheromas, plaque rupture, and microcalcifications at micrometer resolution; correlation studies with circulating trimethylamine-N-oxide (TMAO) show that elevated TMAO correlates positively with lipid arc and lipid volume index, suggesting that gut microbiota metabolites may participate in vulnerability formation by affecting plaque lipid burden (182). Whether imaging indicators can serve as surrogate endpoints for senescence-related plaque vulnerability nonetheless faces unresolved questions: standardization across imaging modalities, quantitative correspondence between imaging features and histological senescence markers (e.g., p16, p21, SA-β-gal), and interference of metabolic disorders with imaging signals in diabetic patients (183). Future research should build a trinity of imaging-molecular-histological validation systems and clarify the predictive validity of imaging surrogate endpoints through large-sample longitudinal cohorts, thereby providing reliable non-invasive tools for clinical plaque vulnerability assessment (184).

### 7.2 Evaluation of therapeutic strategies targeting senescent VSMCs

Therapeutic strategies targeting senescent VSMCs are moving from proof of concept to preclinical evaluation, with senolytic drugs that eliminate senescent cells being the most representative intervention. Intermittent dasatinib plus quercetin (D+Q) treatment reduces senescent cell markers in the aortic media of aged or hypercholesterolemic mice and improves vasomotor function and vascular calcification (185). The non-selective clearance mechanism of these drugs, however, poses potential toxicity to normal tissues—navitoclax-based BCL-2 inhibition is limited by thrombocytopenia due to on-target platelet BCL-xL dependence (186). Senescent VSMCs themselves exhibit anti-apoptotic properties, such as upregulated Bcl-2 expression, which provides a theoretical target for BCL-2 inhibitors but also raises the risk of off-target effects on normal cells (187). Developing delivery systems that precisely target senescent VSMCs within plaques has therefore become a key direction to overcome this bottleneck. Drug delivery systems based on nanoparticles conjugated with anti-SMα-actin antibodies are currently under development; by recognizing VSMC-specific markers, they aim to deliver senolytic drugs precisely to the plaque microenvironment, improving efficacy while reducing systemic toxicity (188). This strategy is expected to achieve “precise clearance” and provide a safer feasibility route for the clinical application of senolytic therapy.

Beyond direct elimination of senescent cells, alternative intervention strategies offer advantages that do not depend on cell death, creating new possibilities for reversing senescence while preserving vascular wall integrity. Forced cell cycle re-entry aims to reverse the growth arrest state of senescent cells, but its potential carcinogenic risk warrants caution (120). SASP-neutralizing antibodies block the adverse effects of the senescence-associated secretory phenotype on surrounding tissues by targeting pro-inflammatory factors (e.g., IL-6 and IL-8) secreted by senescent cells, alleviating chronic inflammation and plaque progression (130, 154). Restoring autophagic flux is another intervention route attracting considerable attention: extensive evidence indicates that autophagy deficiency is a key mechanism driving VSMC senescence, and restoring autophagic activity can effectively delay the senescence process (6, 153). Metformin, for example, enhances autophagic flux by promoting autophagosome-lysosome fusion, suppresses angiotensin II-induced VSMC senescence, and improves arterial stiffness and vascular structure (153). Fisetin likewise restores autophagic activity by activating PPARγ or inhibiting the mTORC2-FoxO3a signaling pathway (189, 190). Simvastatin improves mitochondrial dysfunction in senescent VSMCs and reduces SASP secretion by inhibiting HMG-CoA reductase, exhibiting a “senostatic” (suppressing the senescent phenotype) rather than “senolytic” (eliminating senescent cells) effect (191). The core advantage of these non-clearing strategies lies in modulating intrinsic cellular homeostatic mechanisms (e.g., autophagy and mitochondrial quality control) rather than directly killing cells; they are therefore theoretically less damaging to normal tissues and safer for clinical translation. In the future, combining precise delivery technologies with these alternative intervention strategies may yield more efficient and safer anti-atherosclerotic approaches.

Among the strategies currently under investigation, those that modulate intrinsic cellular homeostatic mechanisms—rather than directly eliminating senescent cells—appear to hold the most immediate translational potential for diabetic patients with atherosclerotic cardiovascular disease. This assessment rests on several considerations. First, senolytic drugs such as dasatinib plus quercetin or navitoclax, although effective in reducing plaque senescent cell burden in preclinical models (154, 188), carry substantial off-target toxicity because their pro-apoptotic mechanism is not VSMC-specific; this concern is amplified in diabetic patients who often have comorbidities affecting the hematopoietic system and gastrointestinal tract. Second, non-clearing “senostatic” strategies that restore autophagic flux—such as metformin, which enhances autophagosome-lysosome fusion and suppresses angiotensin II-induced VSMC senescence (153), or fisetin, which activates PPARγ and inhibits mTORC2-FoxO3a signaling (189, 190)—target pathways that are already implicated in diabetic vascular complications, and metformin in particular has decades of clinical safety data in diabetic populations. Third, strategies that address diabetic-specific drivers—such as FGF21 overexpression alleviating VSMC senescence through the SYK-NLRP3-PPARγ-catalase pathway (44), mitochondria-targeted antioxidant nanosystems reducing high-glucose-induced oxidative stress (25), or natural compounds such as rutin that attenuate VSMC premature senescence in diabetic atherosclerosis models (26)—directly target the hyperglycemic milieu that drives VSMC senescence in the first place. On balance, we consider that autophagy-restoring and diabetic-specific mechanistic interventions—particularly those with existing clinical safety data—are likely to be more readily translatable than broad senolytic approaches, although precise delivery systems that target senescent VSMCs within plaques remain a critical prerequisite for any strategy to reach the clinic.

## 8 Limitations of current research and future directions

### 8.1 Major challenges in experimental models and study design

Animal models of diabetic atherosclerosis fall short of faithfully mirroring human VSMC senescence. The most commonly used models (ApoE−/− and LDLR−/− mice combined with STZ induction, or db/db background models) reproduce part of the features of hyperglycemia and atherosclerosis but remain insufficient to capture the complexity of human VSMC senescence. Species differences are the foremost issue: mice and humans diverge fundamentally in telomere length regulation, in the relative reliance on p53-p21 versus p16-Rb signaling, and in SASP composition, so senescence marker data from mouse models do not extrapolate readily to humans. Different laboratories also employ widely varying high-fat diet formulations, STZ dosing regimens, and modeling durations, producing marked inconsistencies in reported senescence markers (e.g., SA-β-gal positivity rates, p16/p21 expression levels). ApoE−/− mice on a high-fat diet exhibit vascular resistance to platelet-derived growth factor-β (PDGFB) reminiscent of human aging, yet whether this model fully recapitulates the contractile-to-synthetic VSMC conversion accompanied by senescence remains to be validated. Although db/db background models develop hyperglycemia spontaneously, the metabolic disturbances arising from leptin receptor deficiency differ from human type 2 diabetes, because leptin or leptin receptor deficiency is not an important contributor to human T2DM (192).

Most existing studies are cross-sectional, assessing senescence markers in plaque tissue or cultured cells at a single time point and lacking dynamic tracking of when senescent VSMCs appear. Atherosclerosis is a chronic, progressive process; from intimal thickening and lipid deposition to fibrous cap formation and plaque rupture, the VSMC phenotypic state and functional significance may differ markedly across stages (6, 193). Cross-sectional designs cannot tell whether senescence drives plaque formation or follows from it, nor can they establish the precise causal role of senescent cells in plaque progression. scRNA-seq has in recent years become a powerful tool for dissecting VSMC heterogeneity, yet most available studies provide only single-time-point snapshots (130). In Takayasu arteritis research, combined bulk RNA-seq and scRNA-seq analysis showed that VSMCs display premature senescence features and actively participate in vascular inflammation (130). Without temporal data, it is difficult to determine whether VSMC senescence is an early driving event or a late accompanying phenomenon of plaque formation, seriously constraining the design of senescence-targeted intervention strategies.

*In vitro* high-glucose induction models deviate from the true diabetic environment *in vivo* on multiple fronts, and this gap is the main obstacle to translating laboratory findings into *in vivo* validation. First, *in vitro* culture systems lack the mechanical signal of blood flow shear stress; disturbed flow and vascular stiffness are known to drive VSMC phenotypic reprogramming in atherosclerosis (194). Second, they cannot simulate the complex interaction networks between VSMCs and immune cells such as endothelial cells, macrophages, and T cells in the vascular wall. Exosomes from high-glucose-stimulated endothelial cells induce VSMC senescence through the circRNA-0077930-miR-622-Kras ceRNA network, yet traditional monolayer cultures cannot fully reproduce this intercellular communication (65). Lipopolysaccharide-induced senescent macrophages similarly aggravate VSMC calcification and senescence through IFITM3, a paracrine effect often overlooked in *in vitro* high-glucose models (73). Third, *in vitro* culture lacks the three-dimensional structure of the extracellular matrix, and matrix proteins such as COMP suppress VSMC senescence by regulating p53, p21, and p16 expression (195). In addition, *in vitro* high-glucose stimulation typically uses constant glucose concentrations (e.g., 20–25 mmol/L), which do not match the dynamic nature of *in vivo* glycemic fluctuations and omit the accumulation of diabetes-related metabolites such as AGEs (54, 196). These model deficiencies help explain why anti-senescence targets identified *in vitro* (e.g., MMP14, FOS) often fail to yield consistent results in *in vivo* validation (118).

When evaluating the evidence for VSMC senescence in diabetic atherosclerosis, it is useful to distinguish three experimental systems that differ substantially in their translational relevance. *In vitro* studies—most commonly high-glucose-treated cultured VSMCs—have provided detailed mechanistic insights into oxidative stress, DNA damage, and SASP regulation, yet they lack the mechanical shear stress, three-dimensional extracellular matrix, and intercellular signaling networks of the vascular wall (194, 195). *In vivo* preclinical studies, predominantly ApoE−/− or LDLR−/− mice with STZ-induced diabetes or db/db models, capture systemic metabolic complexity and plaque-level pathology but are constrained by species differences in telomere regulation, p53-p21 versus p16-Rb pathway reliance, and SASP composition (119, 120). Human evidence remains relatively sparse: Matthews et al. demonstrated telomere-based senescence in VSMCs from human atherosclerotic plaques, but their study was not stratified by diabetes status (4), and most single-cell transcriptomic datasets have not yet been analyzed separately for diabetic versus non-diabetic plaques (12). We suggest that mechanistic claims derived from *in vitro* work should be validated in appropriate *in vivo* models, and *in vivo* findings should ultimately be anchored to human plaque data before clinical translation is considered.

### 8.2 Standardization and reproducibility issues

A core challenge in senescent VSMC research is the lack of standardization and reproducibility in detection methodology. Detection protocols for senescence markers differ substantially across laboratories, directly affecting the reliability and comparability of results. SA-β-gal, the most classical senescence marker, illustrates the problem: detection results depend heavily on pH control during staining. SA-β-gal activity is optimal at pH 6.0, but laboratories lack unified standards for buffer preparation, incubation time, and temperature control, leading to frequent false-positive or false-negative results (119, 197). Immunohistochemical detection of p16Ink4a and p21 faces analogous problems: minor differences in antigen retrieval conditions (e.g., retrieval solution pH, heating temperature and time) as well as antibody concentration and incubation time can significantly alter staining intensity and background signal, affecting judgments of VSMC senescence status (120). Inconsistent quantitative methods compound the difficulty: the counting standards for SA-β-gal-positive cells and the semi-quantitative analysis of p16 protein expression (e.g., Western blot band gray values) lack recognized thresholds, making direct comparison between studies difficult (151, 198).

At the data analysis level, particularly for scRNA-seq and transcriptomic bioinformatics pipelines, the absence of cross-study benchmarking is another key bottleneck. The scRNA-seq workflow involves multiple steps: data quality control, normalization, dimensionality reduction, clustering, and identification of differentially expressed genes. Multiple algorithms exist for each, and different dimensionality-reduction methods yield substantially different downstream representations (199). The selection of senescence gene sets and scoring algorithms (e.g., “senescence scores” or “SASP scores”) also differs widely across studies. This heterogeneity in analysis strategies means that even when the same dataset is analyzed, different teams may reach different biological conclusions (199).

Establishing consensus guidelines and standard operating procedures (SOPs) is therefore urgent. Researchers should also be encouraged to report experimental conditions, antibody information, data analysis parameters, and code in detail when publishing, to improve transparency and reproducibility. Only through such systematic standardization can VSMC senescence research advance from descriptive observation toward mechanistic validation and clinical translation.

### 8.3 Future technical evolution and multi-omics integration prospects

The rapid development of single-cell multi-omics technologies has opened new routes for the precise identification of senescent VSMCs. Traditional studies rely largely on population-level transcriptomic sequencing, which cannot resolve the molecular features of senescent subpopulations against a background of cellular heterogeneity. With the maturation of technologies such as CITE-seq and Multiome, researchers can now capture multidimensional information (transcriptome, chromatin accessibility, and surface protein expression) simultaneously at single-cell resolution, enabling epigenetic-transcriptomic-protein-level characterization of senescent VSMCs. This advantage is particularly critical in diabetic atherosclerosis: high-glucose conditions drive VSMC senescence through multiple signaling pathways (e.g., the ChREBP/TXNIP/AKT axis, STING1 pathway, and SIRT1 downregulation) (38, 51, 155), whose activation states differ significantly across cell subpopulations; only single-cell multi-omics technologies can resolve such heterogeneity precisely. Integrated multi-omics analysis has also demonstrated great potential for revealing senescence regulatory networks. For example, integrating CUT&Tag and RNA-seq data elucidated the epigenetic mechanism by which NR4A3 regulates vascular calcification through histone lactylation modification (200); combined transcriptomic and metabolomic analysis revealed how CTSD influences VSMC phenotypic conversion by suppressing the glycolytic pathway (54). These examples show that multi-omics integration can establish causal links across molecular levels, providing a solid data foundation for constructing high-dimensional senescence scoring systems. As spatial multi-omics technologies mature, it will become possible to resolve the transcriptomic, proteomic, and metabolomic features of senescent VSMCs within the same tissue region simultaneously, greatly enhancing understanding of the interactions between senescent cells and the surrounding microenvironment and providing more biologically faithful technical support for identifying senescent VSMCs in diabetic atherosclerosis.

Artificial intelligence (AI) and deep learning are reshaping the paradigm of senescent cell pathological identification. Traditionally, identification of senescent cells relies on histological methods such as SA-β-gal staining and p16Ink4a immunohistochemistry, whose interpretation depends heavily on pathologist experience and suffers from subjectivity and low throughput. In recent years, image analysis algorithms based on deep convolutional neural networks have achieved preliminary success in automatically identifying SA-β-gal-positive and p16Ink4a-positive cells in pathological images, enabling high-throughput, standardized quantitative analysis. Differences in staining protocols, microscope platforms, and image acquisition parameters across laboratories, however, limit the generalization performance of models. Domain adaptation and transfer learning offer an effective remedy. Pre-training models on large-scale diverse datasets and fine-tuning them for specific staining protocols and imaging platforms can substantially improve model robustness under different experimental conditions. This technical route complements machine learning methods in bioinformatics: existing studies have used algorithms such as LASSO regression and SVM-RFE to screen key genes closely related to diabetic vascular senescence from transcriptomic data (e.g., TFB1M, FOXRED2, LY75) (158), while others have identified MMP14 and FOS as core regulatory genes of VSMC senescence in diabetic kidney disease through combined random forest, LASSO, and SVM-RFE strategies (118). The successful application of these computational methods suggests that fusing deep learning image recognition with machine learning omics analysis may construct a full-chain senescent VSMC identification system from molecular phenotype to histopathology. The interpretability of AI models remains a key challenge. In clinical translation, models must not only output accurate identification results but also provide interpretable pathological evidence to enhance clinicians’ trust. In the future, pathological image analysis models incorporating attention mechanisms and explainable AI technologies will provide visual evidence while improving identification accuracy, accelerating their practical application in diabetic atherosclerosis research and clinical diagnosis.

Spatial multi-omics technologies provide powerful technical support for comprehensively characterizing the molecular landscape of senescent VSMCs. Traditional omics studies usually require tissue homogenization, inevitably losing the spatial location information of cells, whereas the functional state of senescent cells is closely tied to their local microenvironment. The integrated application of spatial transcriptomics, spatial proteomics, and spatial metabolomics makes it possible to obtain spatial distribution information at multiple molecular levels from the same tissue section. In diabetic atherosclerotic plaques, senescent VSMCs tend to aggregate in specific regions (e.g., fibrous cap and around calcified foci) and form complex interaction networks with the local inflammatory microenvironment (119). Spatial multi-omics analysis allows researchers to pinpoint associations between the molecular features of senescent VSMCs and their spatial neighbors, revealing how senescent cells influence surrounding cells through paracrine mechanisms such as SASP factors. Metabolic reprogramming is another important feature of VSMCs (glycolysis, fatty acid oxidation, and amino acid metabolism all change significantly during vascular disease [78]), and spatial metabolomics can detect the distribution of these metabolites *in situ*, providing direct evidence for understanding the energy metabolism adaptation of VSMCs. Integrating spatial transcriptomic and metabolomic data can construct metabolic-transcriptional regulatory networks of senescent VSMCs and identify key metabolic nodes driving the senescent phenotype. Combined with proteomic data (e.g., detecting the distribution of senescence-associated secreted proteins through spatial proteomic technologies), a comprehensive senescence scoring system covering transcription, translation, and metabolism can be established. This high-dimensional system can not only identify senescent VSMC subpopulations more accurately but also evaluate the regulatory effects of different intervention strategies (e.g., SIRT1 activators, FGF21 overexpression, or natural compounds such as rutin and astaxanthin) on the senescence process (26, 29, 44, 201), providing molecular-level decision-making evidence for precision treatment of diabetic atherosclerosis. As the resolution and throughput of spatial multi-omics technologies improve and bioinformatics analysis methods are refined, this technical route is expected to move from laboratory research to clinical translation within the next few years.

## 9 Conclusion

Taken together, the evidence indicates that identification and functional dissection of senescent VSMCs in diabetic atherosclerosis has moved beyond single-marker dependence into an era of multidimensional, multi-omics integration. Cell cycle inhibitors, SA-β-gal, and SASP factors each carry value, but used alone they all suffer from inherent deficiencies in sensitivity and specificity; future preclinical research and translational applications must therefore establish standardized determination systems based on multi-marker combinations. Advances in transcriptomics and single-cell technologies have enabled us to reveal the heterogeneity of senescent smooth muscle cells at single-cell resolution. The differential distribution of distinct subpopulations across the fibrous cap, lipid core, and neovascularization regions provides key molecular-level evidence for understanding how the diabetic hyperglycemic environment accelerates the senescent phenotype and promotes plaque instability. The methodological field still faces severe challenges: significant deviations exist between *in vitro*-induced senescence models and the true *in vivo* pathological state, and detection protocols adopted by different laboratories lack unified quality control standards, directly limiting the comparability of research results. Establishing cross-platform consensus analysis pipelines and promoting multicenter collaborative groups to share high-quality datasets are therefore urgent priorities for improving identification accuracy. In terms of translational applications, senolytic therapies targeting senescent cells, combined with precise delivery systems such as nanocarriers, have shown encouraging preclinical potential, but key obstacles remain, including insufficient safety assessment and unresolved off-target effects. Future research should prioritize three directions: establishing senescence cell threshold standards based on multi-omics integration; accelerating the clinical validation of biomarkers in prospective cohorts; and promoting the standardization of data and methods through open collaborative mechanisms. Only in this way can the molecular discoveries of basic research be translated into precise intervention strategies accessible to patients with diabetic atherosclerosis.
